# Transcriptomic Analysis of Shiga Toxin-Producing *Escherichia coli* FORC_035 Reveals the Essential Role of Iron Acquisition for Survival in Canola Sprouts and Water Dropwort

**DOI:** 10.3389/fmicb.2018.02397

**Published:** 2018-10-08

**Authors:** Hongjun Na, Yeonkyung Kim, Dajeong Kim, Hyunjin Yoon, Sangryeol Ryu

**Affiliations:** ^1^Department of Food and Animal Biotechnology, Department of Agricultural Biotechnology, Research Institute of Agriculture and Life Sciences, Seoul National University, Seoul, South Korea; ^2^Department of Molecular Science and Technology, Department of Applied Chemistry and Biological Engineering, Ajou University, Suwon, South Korea; ^3^Center for Food and Bioconvergence, Seoul National University, Seoul, South Korea

**Keywords:** enterohemorrhagic *Escherichia coli* FORC_035, canola sprouts, water dropwort, iron acquisition, gallium

## Abstract

Enterohemorrhagic *Escherichia coli* (EHEC) is a foodborne pathogen that poses a serious threat to humans. Although EHEC is problematic mainly in food products containing meat, recent studies have revealed that many EHEC-associated foodborne outbreaks were attributable to spoiled produce such as sprouts and green leafy vegetables. To understand how EHEC adapts to the environment in fresh produce, we exposed the EHEC isolate FORC_035 to canola spouts (*Brassica napus*) and water dropwort (*Oenanthe javanica*) and profiled the transcriptome of this pathogen at 1 and 3 h after incubation with the plant materials. Transcriptome analysis revealed that the expression of genes associated with iron uptake were down-regulated during adaptation to plant tissues. A mutant strain lacking *entB*, presumably defective in enterobactin biosynthesis, had growth defects in co-culture with water dropwort, and the defective phenotype was complemented by the addition of ferric ion. Furthermore, gallium treatment to block iron uptake inhibited bacterial growth on water dropwort and also hampered biofilm formation. Taken together, these results indicate that iron uptake is essential for the fitness of EHEC in plants and that gallium can be used to prevent the growth of this pathogen in fresh produce.

## Introduction

Enterohemorrhagic *Escherichia coli* (EHEC) is a virulent foodborne pathogen that causes outbreaks associated with serious health problems worldwide (Sperandio and Nguyen, 2012). It causes bloody diarrhea, abdominal cramps, and hemolytic uremic syndrome in human, which may be life-threatening, especially in young children and the elderly (Karmali et al., 1983; Karmali, 1989; Griffin and Tauxe, 1991). EHEC is commonly found in animal-based foods made with beef, pork, or poultry (Meng and Doyle, 1997). However, fresh produce (e.g., lettuce, spinach, alfalfa sprouts) has been identified as another notable source of EHEC outbreaks (Taormina et al., 1999; Sivapalasingam et al., 2004). The increase in number of outbreaks associated with fresh produce has been attributed in part to increased consumption of fruits and vegetables in recent decades (Lynch et al., 2009). Furthermore, fresh produce is generally consumed without thermal treatment, which is likely to aggravate the occurrence of produce-associated foodborne diseases (Herman et al., 2015).

A number of studies have shown that many foodborne pathogens can survive and replicate in plants, taking advantage of plant-derived amino acids and carbon compounds as nutrients (Cooley et al., 2003; Jablasone et al., 2005; Schikora et al., 2008; Deering et al., 2012). The interactions between bacterial pathogens and their host plants in the context of unfavorable plant conditions including desiccation, UV radiation, and plant immune responses have also been investigated extensively (Shi et al., 2007; Erickson, 2012; Fink et al., 2012). Interestingly, bacterial determinants required for persistence in plants are variable depending on bacterial genomic features and reservoir host plants (O’brien and Lindow, 1989; Heaton and Jones, 2008; Junker et al., 2011). Therefore, to decipher the mechanisms of persistence and replication of foodborne pathogens in plants, the behavior of diverse bacterial pathogens in contact with a variety of plants should be documented. From a food safety and hygiene perspective, although any produce has the potential to carry foodborne pathogens in the field or during post-harvest management, it is critical to understand bacterial survival strategies in fresh produce, which is consumed without thermal treatments.

Recent studies of plant–microbe interactions have been biased toward lettuce, spinach, and tomato, which are associated with foodborne disease outbreaks (Carey et al., 2009; Kyle et al., 2010). To extend our understanding of the interactions between foodborne bacteria and plants, we investigated canola sprouts (*Brassica napus*) and water dropwort (*Oenanthe javanica*) in this study. Sprouted seeds of plants like canola are cultivated using warm water and therefore provide favorable niches for growth of pathogens such as *Salmonella enterica* and *E. coli* O157:H7 (Andrews et al., 1982; Taormina et al., 1999; Erickson, 2012). A multistate outbreak of EHEC occurred in the United States in 1997 because of alfalfa sprout contamination (Breuer et al., 2001). Water dropworts are members of the genus *Oenanthe* in the plant family Apiaceae and the species *O. javanica* is mostly consumed raw in eastern Asia. Water dropwort is usually cultivated in flooded soil and is prone to contamination with sewage containing bacterial pathogens.

Transcriptome analysis can lead to a systematic understanding of bacterial responses to environmental changes such as contact with plant tissues (Schenk et al., 2012). In this study, to gain insight into the interaction between fresh produce and EHEC, we analyzed the transcriptome of EHEC FORC_035 when exposed to canola sprouts and water dropwort using an RNA-Sequencing (RNA-Seq) approach, as this provides higher resolution data than microarrays or related techniques (Matkovich et al., 2010). FORC_035, a strain isolated from kimchi in South Korea, possesses multiple genes encoding virulence factors including Stx2 and hemolysin. It causes attaching and effacing (A/E) lesions, and has the potential to cause severe diseases in humans when consumed in the form of contaminated fresh produce. Comprehensive transcriptome analysis of FORC_035 in contact with plants provided informative data for unraveling the interaction between pathogens and plants and revealed that iron acquisition was critical for EHEC survival in plants. Iron is an essential element for all living organisms, including bacteria. Therefore, bacteria possess a variety of complex systems for iron acquisition and utilization (Doherty, 2007). Among multiple iron uptake systems, siderophore-mediated iron acquisition is the most common form in bacteria (Visca et al., 2002). *E. coli* has been reported to utilize diverse forms of siderophores such as ferrichrome, enterobactin, yersiniabactin, and catecholate that are transported through highly specific proteins encoded by *fhu* operon, *fep* operon, *ybt* operon, and *fiu*/*cir*/*iroN*, respectively (Braun, 2003). The role of iron in bacterial survival and growth has been extensively investigated in bacterial pathogens that encounter iron starvation stress upon entering their hosts (Skaar, 2010). As a critical growth determinant in plants, we further investigated the role of iron in *E. coli* adaptation to plant environments.

## Materials and Methods

### Bacterial Strains and Culture Conditions

The EHEC isolate strain FORC_035 and isogenic mutant derivatives used in this study are described in **Table 1**. FORC_035 is EHEC (O8:H30) producing Stx2 and hemolysin E. FORC_035 strain was isolated from kimchi by the Incheon Institute of Health and Environment (Incheon, South Korea). The genome sequence of FORC_035 was deposited in the database of the Foodborne pathogen Omics Research Center^1^. Unless stated, all bacterial strains were grown in Luria-Bertani (LB) medium at 37°C, and the following antibiotics were added if needed: kanamycin (25 μg/mL) and ampicillin (100 μg/mL). The selective medium of tryptone bile X-glucuronide (TBX, Oxoid, Cambridge, United Kingdom) was used to enumerate FORC_035 populations. As a nutrient-limitation condition, M9 minimal medium supplemented with 0.4% glucose was used. Fe_2_(SO_4_)_3_ and Ga(NO_3_)_3_ (Sigma-Aldrich, St. Louis, MO, United States) at pH 7.0 in sterile water were added to bacterial cultures to examine the effect of iron on bacterial growth.

**Table 1:** Bacterial strains and plasmids used in this study.

Strain or plasmid	Genotype or relevant characteristics^a^	Reference
**Strains**		
FORC_035	Enterohemorrhagic *Escherichia coli* isolate O8:H30, *stxII*, isolated by Incheon Institute of Health and Environment (Incheon, South Korea)	Foodborne Pathogen Omics Research Center
Δ*entB*	FORC_035 Δ*entB*	This study
**Plasmids**		
pKD13	FRT Km^R^ FRT PS1 PS4 *oriR6Kγ*;Ap^R^	Datsenko and Wanner, 2000
pKD46	P_BAD_-*gam*-*beta*-*exo oriR101 repA101*^ts^;Ap^R^	Datsenko and Wanner, 2000
pCP20	*cI*857λP_R_*flp oripSC101*^ts^;Ap^R^ Cm^R^	Datsenko and Wanner, 2000

*^a^Ap^R^, ampicillin resistant; Km^R^, kanamycin resistant; Cm^R^, chloramphenicol resistant.*

To study the effect of two siderophores, enterobactin and yersiniabactin, Δ*entB* (deletion mutant of *entB* encoding enterobactin synthase component B) and Δ*ybtS* (deletion mutant of *ybtS* encoding yersiniabactin biosynthesis salicylate synthase) were constructed. An isogenic mutant of FORC_035, Δ*entB*, was constructed using the λ Red recombination system. PCR products containing a kanamycin resistance gene were generated using pKD13 as a template (Datsenko and Wanner, 2000; Murphy and Campellone, 2003). For homologous recombination, PCR primers were designed to introduce nucleotides identical to the flanking regions of *entB* at each end of the PCR products (**Supplementary Table S1**). FORC_035 strain harboring the Red helper plasmid pKD46 was grown in the presence of L-arabinose (50 mM) at 30°C with shaking (220 rpm) until the OD_600_ reached 0.6. Bacterial cells were then centrifuged (10,000 × *g*, 5 min, 4°C), resuspended in ice-cold water, and transformed by electroporation (Bio-Rad Laboratories, Hercules, CA, United States). Recombinant cells were selected by kanamycin resistance (Jandu et al., 2009), and subjected to diagnostic PCR using the primers listed in **Supplementary Table S1**. The kanamycin resistance cassette was subsequently removed using the FLP recombinase of pCP20 (Cherepanov and Wackernagel, 1995). A Δ*ybtS* mutant strain was also constructed in the same way using ybtS-Lambda-F/ybtS-Lambda-R primer set (**Supplementary Table S1**).

### Preparation of Canola Sprouts and Water Dropwort

Canola sprouts (*Brassica napus*) and water dropwort (*Oenanthe javanica*) were purchased from commercial markets in Seoul, South Korea. To prepare water dropwort, only stems around 15 cm in length were cut into pieces, because these are more likely to be contaminated by bacteria than other parts of the plant. Five grams of each plant were washed using 50 mL of sterile water three times with agitation (220 rmp) for 10 min each time prior to contact with bacteria.

### *In planta* Assay

To prepare bacterial total RNA after contact with plants, the FORC_035 strain was cultured in LB until OD_600_ reached 1.0, then bacterial cells were washed twice using M9 minimal medium and resuspended in fresh M9 minimal medium (15 mL) at 10^9^ CFU/mL. Five-gram of canola sprouts or water dropwort was added to the bacterial culture and cells were incubated at 37°C with shaking at 220 rpm. The bacterial culture grown in the same condition without adding plants in 20 mL M9 minimal medium was used as a control. At 1- and 3-h post-inoculation, total RNAs were prepared in biological duplicate.

### Total RNA Extraction, Sequencing, and Analysis

One hundred microliter of bacterial cultures was treated with RNAprotect bacterial reagent (Qiagen, Hilden, Germany) to quench RNA degradation, and bacterial total RNAs were isolated using an RNeasy mini kit (Qiagen) according to the manufacturer’s instructions. RNA samples were treated with Ambion Turbo DNA-*free*^TM^ (Ambion, Austin, TX, United States) to remove all genomic DNA. The quantity and quality of total RNA samples were examined using an Agilent 2100 Bioanalyzer (Agilent Technologies, Santa Clara, CA, United States), and RNA samples with RNA integrity numbers (RIN) larger than 9 were used for further analysis (Schroeder et al., 2006). Total RNA samples were stored at −81°C until use.

Five micrograms of total RNA from each sample was used as a starting material and subjected to an rRNA-removal process based on the subtractive hybridization/bead capture system of the Ribo-Zero kit (Epicentre Biotechnologies, Madison, WI, United States). Purified RNA samples were used for mRNA-Seq library construction using the Illumina TruSeq RNA Sample Preparation kit v.2 (Illumina, San Diego, CA, United States). RNA-Seq was performed by two runs of Illumina Hiseq to generate single-end-reads around 100 bp in length. All RNA-Seq data analyzed in this study, including whole transcriptome profiles, are provided as supplementary information files (**Supplementary Tables S2, S3**) and deposited in the Foodborne pathogen Omics Research Center^2^. Using CLRNAseq program (Chunlab, Seoul, South Korea), sequencing reads were mapped to the FORC_035 genome sequence and normalized. Normalization methods employed in RNA-Seq analysis included Reads Per Kilobase of transcript per Million mapped reads (RPKM), Relative Log Expression (RLE), and Trimmed Mean of *M*-value (TMM) (Robinson and Oshlack, 2010; Dillies et al., 2013; Risso et al., 2014) (**Supplementary Table S4**). Because the coefficient of variation (CV) value for the TMM method was lower than those of RPKM and RLE, TMM was used for the normalization of expression level of genes. The *p*-value was calculated using edgeR and the [fold change] value was calculated as [TMM_Freshproduce_/TMM_Control_]. For further experiments, differentially expressed genes (DEGs) with an absolute log_2_ [fold change] larger than 2 were filtered and visualized using the CLRNAseq program (Chunlab, Seoul, South Korea). Clusters of orthologous groups (COG) analysis (Tatusov et al., 1997) was used for functional grouping of all genes of EHEC FORC_035. The proportion of DEGs in each functional group was calculated.

### Heat Map Generation

Heat maps were drawn to graphically visualize global response patterns. Log_2_ [fold change] values were used to determine relative gene expression between FORC_035 only and FORC_035 co-cultured with canola sprouts and water dropwort. Heat maps and hierarchical clusters were then generated using Gitools v2.2.2 (Perez-Llamas and Lopez-Bigas, 2011).

### Quantitative Real-Time RT-PCR (qRT-PCR)

The expression level of specific genes was validated using qRT-PCR as described in Yoo et al. (2017). For all results of qRT-PCR, GAPDH was used as normalization control. The primers used for the detection of genes are listed in **Supplementary Table S5**.

### Construction of pEntB Plasmid

To construct pEntB plasmid expressing *entB* gene, pUHE21-2*lacI*^q^ plasmid was used (Soncini et al., 1995). The *entB* gene was amplified by PCR using the primer set of entB-F-pUHE and entB-R-pUHE (**Supplementary Table S1**). The purified PCR product was inserted between the BamHI and HindIII sites of the vector.

### Bacterial Cell Count Assay for Growth Comparison

Bacterial cells cultured overnight were washed with M9 minimal media and added in 15 mL of fresh M9 minimal media at a 1:100 ratio with or without 5 g of fresh produce. Bacterial viability was examined by plating on TBX selective agar and represented as log(*N*/*N*_0_) (CFU/mL) values, where *N*_0_ is the initial number of cells at 0 h and *N* is the number of cells after 5 h incubation. Ferric sulfate or gallium nitrate at indicated concentrations was added to M9 minimal media when required. For strains harboring empty plasmid or pEntB plasmid, 50 μM of IPTG was used for induction. All experiments were performed in biological triplicate. *P*-value was calculated by Student *t*-test.

### Biofilm Reduction Assay

Bacterial cells cultivated in LB medium overnight were diluted into fresh LB medium (200 μL) at a 1:100 ratio in a polystyrene 96-well plate (SPL, Seoul, South Korea), and grown at 37°C for 48 h without shaking. Ga(NO_3_)_3_ dissolved in sterile water was added at different concentrations when needed. Considering no-observable-adverse-effect level (NOAEL) and acceptable-daily-intake (ADI) values of gallium, Ga(NO_3_)_3_ was used up to 1 mM (Gómez et al., 1992). Biofilm formation was quantified by staining with 0.1% crystal violet for 5 min followed by washing with phosphate buffer (0.1 M, pH 7.4). All dye attached to the biofilm was dissolved with 200 μL of 33% glacial acetic acid, and OD_570_ was measured to quantify the total biofilm mass (Vikram et al., 2010). The assay was performed in biological triplicate, and the *p*-value was calculated by Student *t*-test.

## Results and Discussion

### Comprehensive Transcriptome Analysis of FORC_035 Cultivated With Canola Sprouts or Water Dropwort

**FIGURE 1:**
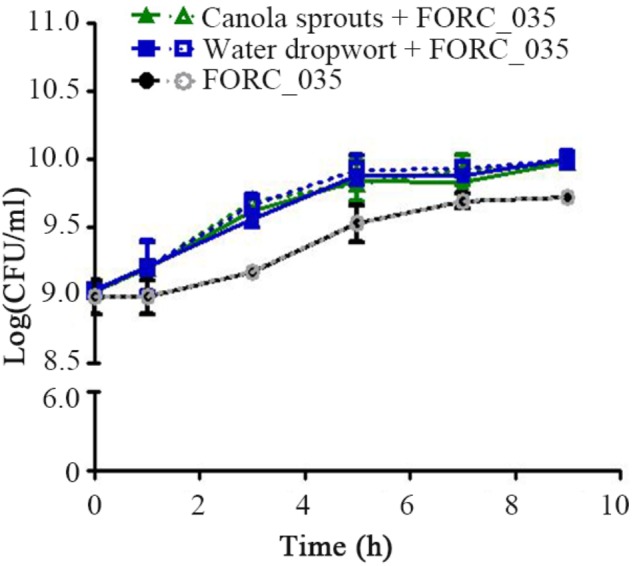
Growth of FORC_035 in M9 minimal media with or without fresh produce. Bacterial cells were inoculated at 10^9^ CFU/mL to fresh M9 minimal media containing canola sprouts or water dropwort, respectively. As a control, FORC_035 was cultivated in M9 minimal medium without plant material. In order to discriminate *E. coli* among indigenous bacteria of plant tissues, culture suspension was diluted and spread onto *E. coli* selective TBX agar (dotted line) and LB agar (solid line), simultaneously. When only plants were incubated, growth of *E. coli* was not detected (**Supplementary Figure S2**). Each symbol indicates the mean value from triplicate measurements.

Bacterial transcriptome analysis is an effective approach to understand the interactions between foodborne pathogens and plants (Kyle et al., 2010; Fink et al., 2012; Landstorfer et al., 2014). In this study, the transcriptome of FORC_035 grown with canola sprouts or water dropwort was analyzed using RNA-Seq technology. Bacterial growth was increased in the presence of plant tissues (**Figure 1**), which was consistent with a previous study showing that bacteria could take advantage of plants-derived nutrients for their growth (Cooley et al., 2003; Jablasone et al., 2005; Schikora et al., 2008; Deering et al., 2012). To understand the bacterial response and adaptations in contact with plants, FORC_035 cells were harvested at 1 and 3 h post-inoculation and their RNAs were isolated for RNA-Seq analysis. RNA-Seq data were acquired, mapped, and normalized as described in the methods (**Supplementary Tables S2–S4**). After mRNA expression levels were compared between plant-exposed samples and not-exposed samples, genes with a log_2_ (fold change) greater than 2 or less than −2 were selected and considered to be DEGs (**Supplementary Figure S1**).

**FIGURE 2:**
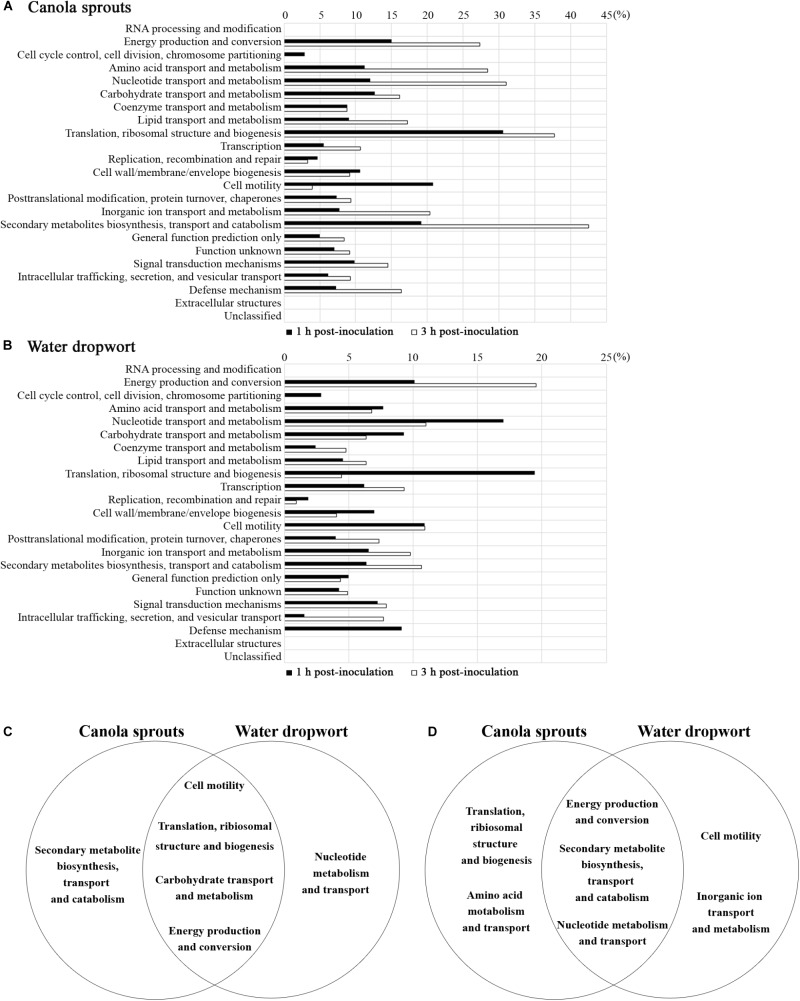
Functional categorization of genes that were differentially regulated upon contact with plants. **(A,B)** Genes with log_2_ [fold change] ≥ 2 or log_2_ [fold change] ≤ –2 in response to canola sprouts **(A)** or water dropwort **(B)** were grouped based on COG. Bars represent the percentages of increased or decreased genes in a given category after 1 h of exposure (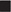) or 3 h of exposure (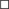). **(C,D)** Venn diagram illustrating functional groups with significant expression changes in contact with plants. Five top functional groups showing differential expressions in response to each plant were sorted at 1 h **(C)** and 3 h **(D)** post-inoculation. Functional groups identified in both of plants in common were placed in the intersections of the sets.

To delineate the physiological changes that occurred in the bacterial cells during contact with plants, 4,977 genes of FORC_035 were categorized according to clusters of orthologous groups (COG) (Tatusov et al., 1997) and the proportion of DEGs in each functional group was calculated (**Figure 2**). In FORC_035 co-cultured with canola sprouts, the expression of 8.14% (405/4,977) and 12.54% (624/4,977) genes changed significantly at 1 and 3 h, respectively. In cells exposed to water dropwort, 5.65% (281/4,977) and 5.67% (282/4,977) of genes exhibited significant differences in their expression at 1 and 3 h, respectively. In both of canola sprouts and water dropwort, co-cultivation for 1 h induced significant expression changes in genes associated with translation, ribosomal structure, and biogenesis (30.56% for canola sprouts and 19.44% for water dropwort), cell motility (20.79% for canola sprouts and 10.89% for water dropwort), energy production and conversion (14.98% for canola sprouts and 10.10% for water dropwort), and carbohydrate transport and metabolism (12.70% for canola sprouts and 9.26% for water dropwort). Meanwhile at 3 h post-inoculation, groups of genes related to secondary metabolites biosynthesis, transport and catabolism (42.55% for canola sprouts and 10.64% for water dropwort), nucleotide transport and metabolism (31.00% for canola sprouts and 11.00% for water dropwort), and energy production and conversion (27.36% for canola sprouts and 19.54% for water dropwort) showed significant differences in their expression in common. Functional groups of genes that showed significant expression changes upon contact with both plants were graphically compared at 1 and 3 h based on COG (**Figures 2C,D**).

### RNA-Seq Analysis Identifies Genes Differentially Expressed in Response to Canola Sprouts or Water Dropwort

Based on their predicted functions, genes up- or down-regulated as a cluster or an operon in response to plant tissues were further defined, because these genes might be involved in bacterial adaptation to plant environments. Genes specifically regulated by exposure to fresh produce and their functions are presented in **Table 2**. Representative genes from each group were validated by qRT-PCR (**Supplementary Figure S3**).

**Table 2:** Genes specifically regulated by exposure to fresh produce.

Gene	Locus tag	Log_2_[fold change]^a^				Function
		1 h		3 h		
		CS^b^	WD^b^	CS	WD	
**Cell motility**						
**Curli**						
*csgF*	FORC35_3421	**−2.63**	**−2.85**	1.22	−1.26	Curli production assembly/transport component CsgF
*csgE*	FORC35_3420	**−2.47**	**−2.86**	**2.93**	1.06	Curli production assembly/transport component CsgE
*csgA*	FORC35_3415	−1.03	−1.31	1.57	1.03	Major curlin subunit precursor CsgA
**Fimbriae**						
*staG*	FORC35_4303	**−2.81**	**−2.65**	1.05	−0.35	Fimbrial protein YadC
*staF*	FORC35_4302	**−2.74**	**−2.31**	1.48	0.09	Fimbrial protein YadK
*staE*	FORC35_4301	**−2.38**	**−2.73**	0.6	−0.48	Fimbrial protein YadL
*staD*	FORC35_4300	**−2.19**	**−2.45**	1.36	−0.66	Fimbrial protein
*fimC*	FORC35_4551	**−4.41**	**−2.29**	0.61	0.06	Fimbrial chaperone protein FimC
*fimD*	FORC35_4550	**−2.08**	**−2.31**	0.39	−0.59	Type 1 fimbriae anchoring protein FimD
*ydeS*	FORC35_4549	−0.89	−1.22	0.23	0.00	Type 1 fimbriae adaptor subunit FimF
*fimH*	FORC35_4547	−0.79	−0.56	0.97	0.10	Mannose-specific adhesin FimH
**Amino acid metabolism and transport**						
**Arg and pro metabolism**						
*astC*	FORC35_3008	**−3.57**	**−3.64**	**6.57**	0.78	Succinylornithine transaminase
*astA*	FORC35_3007	**−3.21**	**−3.11**	**6.11**	−0.37	Arginine *N*-succinyltransferase
*astD*	FORC35_3006	**−3.13**	**−2.84**	**5.52**	−0.60	Succinylglutamic semialdehyde dehydrogenase
*astB*	FORC35_3005	**−3.16**	**−2.54**	**5.6**	−0.27	Succinylarginine dihydrolase
*astE*	FORC35_3004	**−2.93**	**−2.28**	**5.76**	−0.12	Succinylglutamate desuccinylase
*puuB*	FORC35_2444	−1.53	**−2.32**	1.81	−0.37	Gamma-glutamyl-putrescine oxidase
*puuC*	FORC35_2443	−1.80	**−2.36**	**2.01**	−0.77	Gamma-glutamyl-aminobutyraldehyde dehydrogenase
*puuD*	FORC35_2440	−1.76	**−2.44**	**2.69**	0.68	Gamma-glutamyl-GABA hydrolase
**Nitrogen metabolism**						
*narG*	FORC35_2283	**2.07**	**5.29**	0.77	**6.02**	Respiratory nitrate reductase alpha chain
*nark*	FORC35_2282	**2.65**	**6.73**	0.86	**4.35**	Nitrate/nitrite transporter NarK
*narU*	FORC35_2686	**−4.60**	−1.55	**4.58**	**2.90**	Nitrite extrusion protein 2
*narZ*	FORC35_2685	**−3.68**	−1.92	**3.93**	**2.50**	Respiratory nitrate reductase alpha chain
*nary*	FORC35_2684	**−3.16**	−1.48	**3.6**	**2.27**	Respiratory nitrate reductase beta chain
*narW*	FORC35_2683	**−3.63**	−1.62	**4.06**	**2.54**	Respiratory nitrate reductase delta chain
*narV*	FORC35_2682	**−3.45**	−1.59	**3.55**	**2.17**	Respiratory nitrate reductase gamma chain
**Carbohydrate transport**						
**PTS system**						
*sgcC*	FORC35_4568	0.55	1.05	**4.46**	1.90	PTS system, galactitol-specific IIC component
*sgcB*	FORC35_4567	0.01	0.01	**4.40**	**3.12**	PTS system, galactitol-specific IIB component
*sgcX*	FORC35_4566	0.26	0.42	**2.55**	**3.23**	Putative sgc region protein SgcX
**Galactose transport**						
*mglB*	FORC35_1870	0.53	**4.85**	−1.00	**4.66**	Galactose/methyl galactoside ABC transport system, D-galactose-binding periplasmic protein MglB
*ytfQ*	FORC35_4683	−0.22	−0.25	**5.77**	**5.58**	Galactofuranose ABC transporter periplasmic binding protein
*ytfR*	FORC35_4682	0.12	−0.08	**3.66**	**2.90**	Galactofuranose ABC transporter putative ATP binding subunit
*ytfT*	FORC35_4681	0.46	−0.04	**3.96**	1.83	Galactofuranose ABC transporter putative membrane subunit
*yjfF*	FORC35_4680	0.25	0.24	**3.54**	0.50	Galactofuranose ABC transporter putative membrane subunit
**Inorganic ion transport and metabolism**						
**Yersiniabactin cluster**						
*ybtS*	FORC35_2144	1.05	0.86	**−3.78**	0.39	Anthranilate synthase
*ybtX*	FORC35_2143	1.50	1.03	**−3.48**	−0.11	AmpG permease
*ybtQ*	FORC35_2142	1.03	0.23	**−3.38**	−0.02	Inner membrane ABC-transporter YbtQ
*ybtA*	FORC35_2140	0.45	1.00	**−5.59**	**−4.34**	Iron acquisition regulator
*irp2*	FORC35_2139	0.85	0.68	**−4.89**	−1.33	Siderophore biosynthesis non-ribosomal peptide synthetase modules
*irp1*	FORC35_2138	0.10	0.54	**−4.06**	−0.93	Iron acquisition yersiniabactin synthesis enzyme
*ybtU*	FORC35_2137	−0.07	0.77	**−3.33**	−0.41	Thiazolinyl imide reductase in siderophore biosynthesis
*ybtT*	FORC35_2136	0.03	0.5	**−3.76**	−0.58	Yersiniabactin synthetase, thioesterase component
*ybtE*	FORC35_2135	−0.11	0.66	**−3.46**	−0.72	2,3-Dihydroxybenzoate-AMP ligase of siderophore
*fyuA*	FORC35_2134	1.91	0.08	**−5.19**	−1.38	TonB-dependent receptor
**Enterobactin cluster**						
*entD*	FORC35_3892	**3.45**	0.55	**−5.02**	**−5.35**	4′-Phosphopantetheinyl transferase EntD
*fepA*	FORC35_3890	**3.71**	−0.12	**−7.13**	**−5.62**	Ferrienterochelin and colicins receptor
*entE*	FORC35_3878	**2.42**	0.72	**−6.61**	**−3.97**	Enterobactin synthase subunit E
*entB*	FORC35_3877	**3.01**	**2.05**	**−7.61**	**−3.63**	2,3-Dihydroxybenzoate–AMP ligase
*entA*	FORC35_3876	**2.78**	**2.05**	**−6.43**	**−3.75**	2,3-Dihydro-2,3-dihydroxybenzoate dehydrogenase
*entH*	FORC35_3875	**2.53**	1.93	**−6.06**	**−3.98**	Proofreading thioesterase EntH

*^a^[Fold change] was defined as [TMM_Fresh produce_/TMM_Control_]. Means from duplicate experiments. ^b^CS, canola sprouts; WD, water dropwort. ^c^Values with significant expression differences are in bold (log_2_[fold change] > 2, log_2_[fold change] < −2). ^d^Log_2_[fold change] was highlighted as four groups: 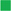 ≤ −2; 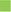 ≤ −1; < 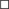 < 1; 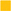 ≥ 1; 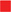 ≥ 2.*

#### (i) Adhesion

In this study, genes encoding various types of fimbriae, including curli and type 1 fimbriae, were down-regulated upon contact with canola sprouts and water dropwort (**Table 2**). Bacteria switch between planktonic and sessile states depending on environmental conditions. When they encounter stresses such as nutrient limitation, they adhere to surfaces and develop extracellular polymeric substances (EPSs) to form a bacterial aggregation called a biofilm (Oh et al., 2007), which enables them to resist various stresses and acquire scarce nutrients (Enos and Taylor, 1996). Bacteria utilize diverse cell surface appendages, including fimbriae, lipopolysaccharides, *O*-antigen capsule, flagellae, and non-fimbrial adhesions for initial attachment to surfaces (Kierek-Pearson and Karatan, 2005). For example, enteric pathogens such as *E. coli* and *Salmonella enterica* possess several different kinds of fimbrial structures and exploit them to colonize salad and alfalfa sprouts (Barak et al., 2005; Fink et al., 2012). In contrast, bacteria favor a planktonic lifestyle under nutrient-rich conditions (Marshall et al., 1971; Brown et al., 1977). Therefore, consistent with the increased growth of FORC_035 in the presence of plants (**Figure 1**), the down-regulation of fimbriae genes in response to plants implies that free-living cells are preferred due to the abundance of nutrients exuded from plant tissues.

#### (ii) Amino Acid and Nitrogen Metabolism

In accordance with the profound expression changes of genes associated with energy production and conversion (**Figure 2**), numerous genes important for amino acid and nitrogen metabolism showed significantly altered expression upon contact with plants, intimating the efflux of nutrients from co-inoculated plant tissues.

For example, genes in the *ast* and *puu* operons, which are required for arginine and proline metabolism, respectively, tended to be induced during adaptation in plant-supplemented environments (**Table 2**): the log_2_ [fold change] was higher at 3 h than at 1 h in both plants. Arginine succinyltransferase (AST) pathway comprises an *ast* operon that allows utilization of arginine as a nitrogen source in *E. coli* (Schneider et al., 1998). Genes in the *puu* operon encode enzymes required for degradation of putrescine, a major polyamine molecule involved in a variety of biological processes, including growth rate control (Tweeddale et al., 1998). The induction of genes involved in arginine and putrescine degradation during bacterial adaptation to plant tissues might be indicative of abundant nitrogen during early exposure, but the depletion of preferable nitrogen sources due to bacterial overgrowth in the medium at later time points. In a similar context, considering that canola sprouts are enriched in arginine and proline (Chung et al., 1989), the overproduction of AST enzymes in canola sprout-supplemented medium at 3 h might be a bacterial strategy to exploit plant arginine as an alternative nitrogen source. Bacteria utilize putrescine not only as a sole nitrogen source, but also as a signaling molecule in response to unfavorable conditions such as oxidative stress, which is a plausible stress in the context of contact with plant tissues (Schneider and Reitzer, 2012). Interestingly, arginine can be converted to putrescine, suggesting that the two pathways are closely related (Schneider et al., 2013).

Bacteria colonizing plants are able to scavenge nitrate, a source of nitrogen in plants (Mantelin and Touraine, 2004), and utilize it as an alternative electron acceptor for energy generation (Bonnefoy and Demoss, 1994). *E. coli* produces two different membrane-bound respiratory nitrate reductases, nitrate reductase A (NRA) and nitrate reductase Z (NRZ), for dissimilative nitrate reduction (Bonnefoy and Demoss, 1994). Transcriptome analysis revealed that the expression of genes encoding NRA (*narG* and *narK*) and NRZ (*narUZYWV*) were both significantly altered during adaptation to a plant environment (**Table 2**); there was an increase in *narGK* expression at both time points, while *narUZYWV* expression decreased at 1 h but increased at 3 h. These results suggest that FORC_035 might operate an aerobic respiration system for shorter exposure times to plant material, but convert to anaerobic respiration for longer exposure times, using nitrate leaked from plant tissues as an electron acceptor.

#### (iii) Carbohydrate Transport

Given the role of sugars and carbohydrates as carbon sources, *E. coli* employs a range of different transporter systems for efficient sugar acquisition, including the phosphotransferase system (PTS) and ATP-binding cassette (ABC) transporter system (Braun, 2003). The genes *sgcCBX, mglB*, *ytfQRT*, and *ytfF* were up-regulated during FORC_035 adaptation to both plant types (**Table 2**). Genes of the *sgc* operon encode a PTS and are involved in uptake and breakdown of pentose and pentitol (Reizer et al., 1996; Tchieu et al., 2001). Products of *mglB*, *ytfQRT*, and *ytfF* comprise ABC transporters for galactopyranose (*mglB*) or galactofuranose (*ytfQRT* and *ytfF*) (Horler et al., 2009). When preferred carbon sources such as glucose are in limited supply, bacteria undergo catabolite repression to consume secondary carbon sources and improve their fitness in response to changes in nutrients (Stülke and Hillen, 1999). *E. coli* deprived of glucose is known to up-regulate the expression of genes required for uptake of alternative sugars such as galactose, ribose, and mannose (Busby and Kolb, 1996; Raman et al., 2005). Up-regulation of genes involved in diverse carbohydrate transport at 3 h post-inoculation in this study suggests that FORC_035 might experience nutrient starvation at 3 h after contact with plants and switch metabolic pathways to utilize alternative carbon sources abundant in plant tissues.

#### (iv) Iron Acquisition

Iron is a critical nutrient for bacterial growth and also important for virulence in pathogenic bacteria (Miethke and Marahiel, 2007). Therefore, bacteria possess many different iron transport systems (Porcheron et al., 2013). *E. coli* also exploits a variety of chelating compounds for iron uptake and siderophores are the primary chelators responsible for ferric iron acquisition (Miethke and Marahiel, 2007). *E. coli* produces redundant siderophores including enterobactin, salmochelin, and aerobactin. In addition, yersiniabactin, a bacterial siderophore found in various pathogenic *Yersinia* species, is also present in several pathogenic *E. coli* strains (Garcia et al., 2011; Johnstone and Nolan, 2015). The complex of ferric ion and siderophore is actively transported across the outer membrane using ATP hydrolysis mediated by the TonB-ExbB-ExbD system (Braun, 2003). Once in the periplasm, periplasmic binding proteins (PBPs) capture ferric-siderophores and deliver them to cytoplasmic membrane ABC transporters to release ferric iron into the cytosol for subsequent utilization as a nutrient (Braun, 2003). Transcriptome analysis revealed a significant change in the expression of genes associated with iron uptake. In particular, the expression of genes required for the production of yersiniabactin and enterobactin siderophores declined during bacterial adaptation to plant tissues (**Table 2**). Taking into account the high binding affinities of siderophores for ferric iron under iron-deficient conditions, decreased expression of siderophore genes at 3 h post-inoculation might be indicative of an abundant supply of iron from plant exudates.

### Enterobactin Is Required for Survival of FORC_035 in Water Dropwort

**FIGURE 3:**
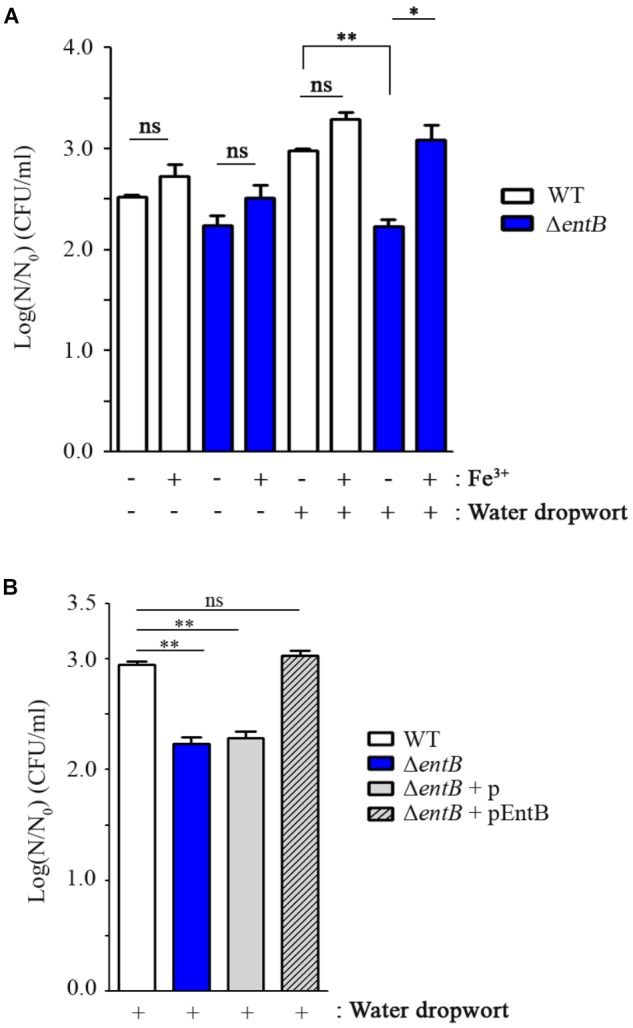
Comparison of bacterial growth between FORC_035 wild-type (WT) and Δ*entB* mutant strains. **(A)** Ferric sulfate (10 μM) was added to M9 minimal media with or without water dropwort. **(B)** Four strains of WT, Δ*entB*, and Δ*entB* with either empty plasmid or pEntB were cultivated in the presence of water dropwort. pUHE21-2*lacI*^q^ plasmid was used and 50 μM of IPTG was used for induction. In both graphs, live bacterial numbers were measured in M9 minimal medium broth supplemented with water dropwort after a 5-h incubation. Viability of bacterial cells was measured by comparing log(*N*/*N*_0_) values, where *N*_0_ is the initial number of cells at 0 h and *N* is the number of cells after a 5-h incubation. The *t*-test was used to evaluate the significance of differences in viability, and significance is indicated as follows: ^∗^*P* < 0.05; ^∗∗^*P* < 0.01; ns, not significant.

Iron is an essential nutrient involved in a variety of biological processes in bacteria, including DNA synthesis, electron transport system, ATP synthesis, and oxygen transport (Andrews et al., 2003; Krewulak and Vogel, 2008). Multiple genes relevant to iron uptake showed an opposing expression tendency between 1 and 3 h post-inoculation; up-regulation at 1 h versus down-regulation at 3 h (**Table 2** and **Supplementary Figure S4**). The expression levels were quantified by qRT-PCR, supporting consistency with the RNA-Seq results (**Supplementary Figure S5**). A similar gene expression pattern was observed when bacterial cells were cultured at room temperature (25°C) in the presence of fresh produce (**Supplementary Figures S6, S7**). These results suggest that FORC_035 might encounter a dynamic change in iron abundance during its adaptation to the plant environment and control its iron uptake ability to improve its fitness. Especially, genes associated with two siderophores, enterobactin and yersiniabactin, were down-regulated by 3 log-fold or more when bacterial cells were exposed to fresh produce for 3 h (**Table 2**). Considering the indispensable roles of iron in numerous biological processes, we evaluated the importance of iron uptake via enterobactin and yersiniabactin during bacterial adaptation to the plants by comparing the growth of Δ*entB* and Δ*ybtS* strains in the presence and absence of plant tissues. Deletion of *entB* did not compromise bacterial growth significantly in M9 minimal medium supplemented with canola sprouts. Taking into account the observation that co-culturing with plant tissues stimulated FORC_035 to alter transcription of diverse genes required for iron transport, including those involved in ferrous ion and ferric citrate transport (**Supplementary Figure S4**), we reasoned that the Δ*entB* strain might be able to circumvent the lack of enterobactin by activating iron transport systems other than enterobactin to acquire iron released from canola sprouts. However, lack of *entB* dampened bacterial growth significantly in the presence of water dropwort, which has a lower iron content than sprouts (Park and Kim, 1996) (**Figure 3A** and **Supplementary Figure S8**). This result suggests that the Δ*entB* strain suffered from a shortage of iron when interacting with water dropwort due to its lack of enterobactin. To test this possibility, ferric sulfate was added to the culture medium at 10 μM as an iron supplement and bacterial growth was compared. As expected, addition of ferric ion complemented the growth defect of the Δ*entB* strain in water dropwort-containing medium (**Figure 3A**). Moreover, the growth defect was complemented with *trans*-encoded *entB* by the pEntB plasmid (**Figure 3B**), indicating that iron uptake via enterobactin is required for the growth of bacterial cells exposed to water dropwort. On the other hand, the mutation on yersiniabactin showed no defects in bacterial growth. These results suggested that enterobactin plays a greater role than yersiniabactin does for iron acquisition. Differential contribution to bacterial growth by enterobactin and yersiniabactin has been reported previously in *Klebsiella pneumonia* (Lawlor et al., 2007).

### Gallium as an Iron Mimetic Inhibits FORC_035 Growth and Biofilm Formation

**FIGURE 4:**
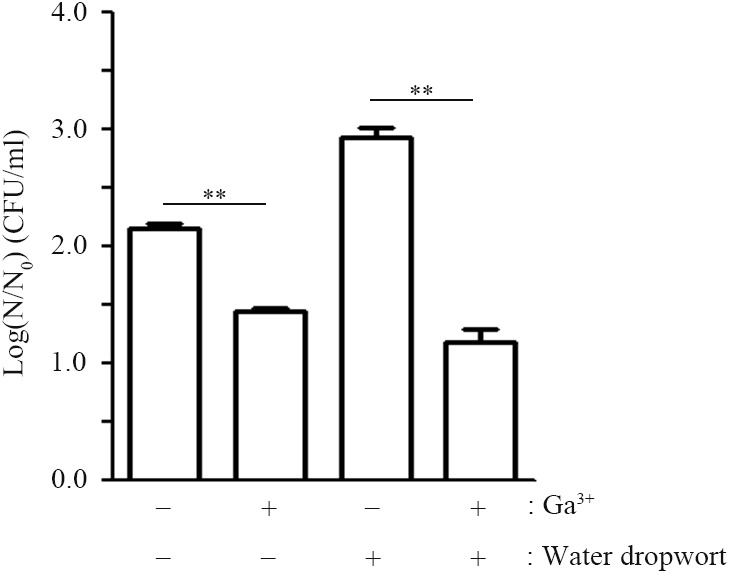
Effect of gallium nitrate on the growth of FORC_035. Wild-type FORC_035 was cultivated in M9 minimal medium broth with or without water dropwort and Ga(NO_3_)_3_ was added to the medium at 1 mM. Bacterial numbers were estimated at 9 h post-inoculation. Note that log(*N*/*N*_0_) is plotted on *y*-axis, where *N*_0_ is the initial number of cells at 0 h and *N* is the number of cells after a 9-h incubation. Asterisks indicate significant differences (^∗∗^*P* < 0.01).

The transition metal gallium (Ga^3+^), which has an ionic radius nearly identical to iron (Fe^3+^), can act as a “Trojan horse” to disturb bacterial iron uptake, as many biologic systems, including bacteria, cannot distinguish between Ga^3+^ and Fe^3+^ (Chitambar and Narasimhan, 1991; Kaneko et al., 2007). Taking into account that the Δ*entB* strain suffering from iron shortage due to its lack of enterobactin slowed down its growth when in contact with water dropwort, disturbance of cellular iron uptake by the addition of gallium could be a promising strategy to control the growth and survival of EHEC FORC_035 on water dropwort. To examine this possibility, FORC_035 in M9 minimal medium containing water dropwort was supplemented with Ga(NO_3_)_3_ at 1 mM and its growth was evaluated (**Figure 4**). FORC_035 co-cultured with water dropwort multiplied faster than FORC_035 grown in the absence of plant tissues, but the addition of gallium led to a 1.7 log reduction in bacterial numbers, indicating that gallium had antimicrobial activity against EHEC FORC_035. Gallium had a growth inhibitory effect regardless of the presence of water dropwort, but gallium exerted greater growth inhibition when bacteria were in contact with water dropwort tissues than when they were not (**Figure 4**). The greater growth inhibition of gallium in the presence of water dropwort was probably because the growth-promoting effect of the plant (**Figure 1**) was abolished by gallium, which functioned as a competitive antagonist of iron in the plant-supplemented environment. Antimicrobial effect of gallium has been observed in other bacteria as well, including *Pseudomonas aeruginosa* and *Mycobacterium tuberculosis* wherein gallium inhibits cellular Fe-dependent metabolic pathways by substituting Fe^3+^ (Olakanmi et al., 2000; Kaneko et al., 2007). In mammalian cells, Ga^3+^ makes up Ga-transferrin complex instead of Fe^3+^, suggesting the possibility that gallium exerts its role by combining with iron-chelating proteins.

**FIGURE 5:**
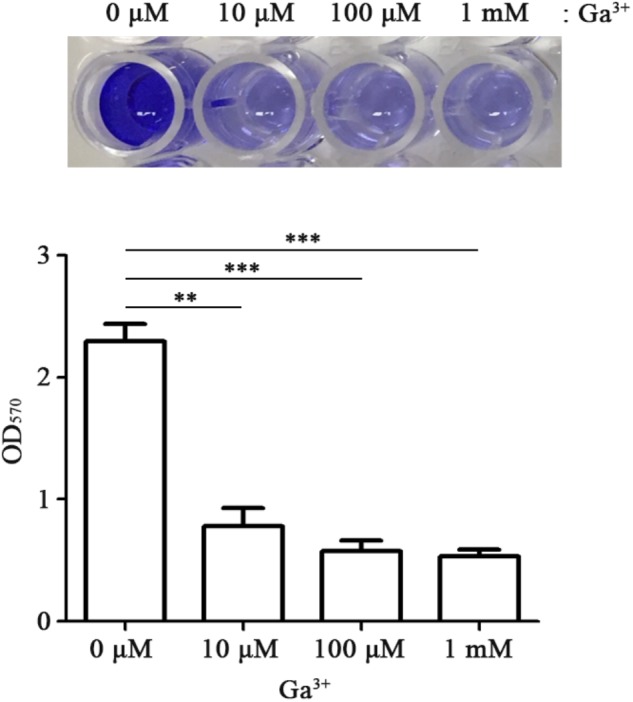
Gallium inhibits biofilm formation of FORC_035. FORC_035 was statically cultivated in LB medium in polystyrene 96-well plates for 48 h. Gallium was added at the indicated concentrations. Biofilm was stained using 0.1% crystal violet **(Top)** and OD_570_ was measured **(Bottom)**. Asterisks indicate significant differences (^∗∗^*P* < 0.01; ^∗∗∗^*P* < 0.001).

Biofilm formation is achieved through a complex process influenced by multiple environmental signals. Among diverse signals, iron can also regulate biofilm formation and this regulation depends on bacterial species. The availability of iron influences the ability of bacteria to form biofilms effectively; high concentrations of iron promote bacterial biofilm development in *E. coli* and *P. aeruginosa* (Banin et al., 2005; Wu and Outten, 2009). Therefore, the influence of gallium on bacterial biofilm formation was investigated. The presence of Ga(NO_3_)_3_ at 10 μM inhibited biofilm formation by threefold without changing the growth of FORC_035 (**Figure 5**). The inhibitory effect of gallium on biofilm formation was not influenced by temperature changes (**Supplementary Figure S9**). Biofilms enable bacteria to resist stressful conditions such as disinfectants and the defense responses of plants and animals, which makes biofilms an important issue to deal with in food hygiene (Srey et al., 2013). Gallium was approved for hypercalcemia treatment by the FDA, and has low toxicity at appropriate doses (Chitambar and Narasimhan, 1991; Gómez et al., 1992). The inhibitory effects of gallium on bacterial growth and biofilm formation suggest that gallium can potentially be used as an antimicrobial agent.

## Conclusion

In conclusion, the transcriptomes of FORC_035 in contact with canola sprouts and water dropwort provided insights into the overall bacterial transcriptional response to plants. There were significant changes in the expression of genes associated with fitness and survival upon co-culture with plant materials. In particular, genes required for iron uptake were found to be critical determinants of the adaptation of EHEC FORC_035 to plants.

## Data Availability

The database of the Foodborne pathogen Omics Research Center (http://forcdb.snu.ac.kr) in which the datasets for this manuscript were deposited are not publicly available due to policy of the institution. Requests to access the datasets should be directed to SR.

## Author Contributions

HN, HY, and SR conceived and designed the experiments. HN, YK, and DK performed the experiments. HN, YK, DK, and HY analyzed the data and wrote the paper. HY and SR revised the manuscript.

## Conflict of Interest Statement

The authors declare that the research was conducted in the absence of any commercial or financial relationships that could be construed as a potential conflict of interest.

## Footnotes

1 http://forcdb.snu.ac.kr/data/genomes

2 http://forcdb.snu.ac.kr/data/transcriptomes

## References

[B1] Andrews S. C. Robinson A. K. Rodríguez-Quiñones F. (2003). Bacterial iron homeostasis. *FEMS Microbiol. Rev.* 27 215–237. 10.1016/S0168-6445(03)00055-X 12829269

[B2] Andrews W. Mislivec P. Wilson C. Bruce V. Poelma P. Gibson R. (1982). Microbial hazards associated with bean sprouting. *J. Assoc. Off. Anal. Chem.* 65 241–248. 7085541

[B3] Banin E. Vasil M. L. Greenberg E. P. (2005). Iron and *Pseudomonas aeruginosa* biofilm formation. *Proc. Natl. Acad. Sci. U.S.A.* 102 11076–11081. 10.1073/pnas.0504266102 16043697 PMC1182440

[B4] Barak J. D. Gorski L. Naraghi-Arani P. Charkowski A. O. (2005). *Salmonella enterica* virulence genes are required for bacterial attachment to plant tissue. *Appl. Environ. Microbiol.* 71 5685–5691. 10.1128/AEM.71.10.5685-5691.2005 16204476 PMC1265987

[B5] Bonnefoy V. Demoss J. A. (1994). Nitrate reductases in *Escherichia coli*. *Antonie Van Leeuwenhoek* 66 47–56. 10.1007/BF00871632 7747940

[B6] Braun V. (2003). Iron uptake by *Escherichia coli*. *Front. Biosci.* 8 s1409–s1421. 12957834 10.2741/1232

[B7] Breuer T. Benkel D. H. Shapiro R. L. Hall W. N. Winnett M. M. Linn M. J. (2001). A multistate outbreak of *Escherichia coli* O157: H7 infections linked to alfalfa sprouts grown from contaminated seeds. *Emerg. Infect. Dis.* 7 977–982. 10.3201/eid0706.010609 11747724 PMC2631892

[B8] Brown C. Ellwood D. Hunter J. (1977). Growth of bacteria at surfaces: influence of nutrient limitation. *FEMS Microbiol. Lett.* 1 163–166. 10.1111/j.1574-6968.1977.tb00605.x

[B9] Busby S. Kolb A. (1996). “The CAP modulon,” in *Regulation of Gene Expression in Escherichia coli*, eds Lin E. C. C. Lynch A. S. (Berlin: Springer), 255–279. 10.1007/978-1-4684-8601-8_12

[B10] Carey C. M. Kostrzynska M. Thompson S. (2009). *Escherichia coli* O157: H7 stress and virulence gene expression on Romaine lettuce using comparative real-time PCR. *J. Microbiol. Methods* 77 235–242. 10.1016/j.mimet.2009.02.010 19248811

[B11] Cherepanov P. P. Wackernagel W. (1995). Gene disruption in *Escherichia coli*: Tc R and Km R cassettes with the option of Flp-catalyzed excision of the antibiotic-resistance determinant. *Gene* 158 9–14. 10.1016/0378-1119(95)00193-A 7789817

[B12] Chitambar C. R. Narasimhan J. (1991). Targeting iron-dependent DNA synthesis with gallium and transferrin-gallium. *Pathobiology* 59 3–10. 10.1159/000163609 1645976

[B13] Chung T. Nwokolo E. Sim J. (1989). Compositional and digestibility changes in sprouted barley and canola seeds. *Plant Foods Hum. Nutr.* 39 267–278. 10.1007/BF01091937 2608636

[B14] Cooley M. B. Miller W. G. Mandrell R. E. (2003). Colonization of Arabidopsis thaliana with *Salmonella enterica* and enterohemorrhagic *Escherichia coli* O157: H7 and competition by *Enterobacter asburiae*. *Appl. Environ. Microbiol.* 69 4915–4926. 10.1128/AEM.69.8.4915-4926.2003 12902287 PMC169118

[B15] Datsenko K. A. Wanner B. L. (2000). One-step inactivation of chromosomal genes in *Escherichia coli* K-12 using PCR products. *Proc. Natl. Acad. Sci. U.S.A.* 97 6640–6645. 10.1073/pnas.120163297 10829079 PMC18686

[B16] Deering A. J. Mauer L. J. Pruitt R. E. (2012). Internalization of *E. coli* O157: H7 and *Salmonella* spp. in plants: a review. *Food Res. Int.* 45 567–575. 10.1016/j.foodres.2011.06.058

[B17] Dillies M.-A. Rau A. Aubert J. Hennequet-Antier C. Jeanmougin M. Servant N. (2013). A comprehensive evaluation of normalization methods for Illumina high-throughput RNA sequencing data analysis. *Brief. Bioinform.* 14 671–683. 10.1093/bib/bbs046 22988256

[B18] Doherty C. P. (2007). Host-pathogen interactions: the role of iron. *J. Nutr.* 137 1341–1344. 10.1093/jn/137.5.1341 17449603

[B19] Enos D. Taylor S. (1996). Influence of sulfate-reducing bacteria on alloy 625 and austenitic stainless steel weldments. *Corrosion* 52 831–842. 10.5006/1.3292075

[B20] Erickson M. C. (2012). Internalization of fresh produce by foodborne pathogens. *Annu. Rev. Food Sci. Technol.* 3 283–310. 10.1146/annurev-food-022811-101211 22243280

[B21] Fink R. C. Black E. P. Hou Z. Sugawara M. Sadowsky M. J. Diez-Gonzalez F. (2012). Transcriptional responses of *Escherichia coli* K-12 and O157: H7 associated with lettuce leaves. *Appl. Environ. Microbiol.* 78 1752–1764. 10.1128/AEM.07454-11 22247152 PMC3298177

[B22] Garcia E. C. Brumbaugh A. R. Mobley H. L. (2011). Redundancy and specificity of *Escherichia coli* iron acquisition systems during urinary tract infection. *Infect. Immun.* 79 1225–1235. 10.1128/IAI.01222-10 21220482 PMC3067483

[B23] Gómez M. Sánchez D. J. Domingo J. L. Corbella J. (1992). Developmental toxicity evaluation of gallium nitrate in mice. *Arch. Toxicol.* 66 188–192. 10.1007/BF01974013 1497482

[B24] Griffin P. M. Tauxe R. V. (1991). The epidemiology of infections caused by *Escherichia coli* O157: H7, other enterohemorrhagic *E. coli*, and the associated hemolytic uremic syndrome. *Epidemiol. Rev.* 13 60–98. 10.1093/oxfordjournals.epirev.a036079 1765120

[B25] Heaton J. C. Jones K. (2008). Microbial contamination of fruit and vegetables and the behaviour of enteropathogens in the phyllosphere: a review. *J. Appl. Microbiol.* 104 613–626. 10.1111/j.1365-2672.2007.03587.x 17927745

[B26] Herman K. Hall A. Gould L. (2015). Outbreaks attributed to fresh leafy vegetables, United States, 1973–2012. *Epidemiol. Infect.* 143 3011–3021. 10.1017/S0950268815000047 25697407 PMC4591532

[B27] Horler R. S. Müller A. Williamson D. C. Potts J. R. Wilson K. S. Thomas G. H. (2009). Furanose-specific sugar transport characterization of a bacterial galactofuranose-binding protein. *J. Biol. Chem.* 284 31156–31163. 10.1074/jbc.M109.054296 19744923 PMC2781514

[B28] Jablasone J. Warriner K. Griffiths M. (2005). Interactions of *Escherichia coli* O157: H7, *Salmonella typhimurium* and *Listeria monocytogenes* plants cultivated in a gnotobiotic system. *Int. J. Food Microbiol.* 99 7–18. 10.1016/j.ijfoodmicro.2004.06.011 15718025

[B29] Jandu N. Ho N. K. Donato K. A. Karmali M. A. Mascarenhas M. Duffy S. P. (2009). Enterohemorrhagic *Escherichia coli* O157: H7 gene expression profiling in response to growth in the presence of host epithelia. *PLoS One* 4:e4889. 10.1371/journal.pone.0004889 19293938 PMC2654852

[B30] Johnstone T. C. Nolan E. M. (2015). Beyond iron: non-classical biological functions of bacterial siderophores. *Dalton Trans.* 44 6320–6339. 10.1039/c4dt03559c 25764171 PMC4375017

[B31] Junker R. R. Loewel C. Gross R. Dötterl S. Keller A. Blüthgen N. (2011). Composition of epiphytic bacterial communities differs on petals and leaves. *Plant Biol.* 13 918–924. 10.1111/j.1438-8677.2011.00454.x 21972888

[B32] Kaneko Y. Thoendel M. Olakanmi O. Britigan B. E. Singh P. K. (2007). The transition metal gallium disrupts *Pseudomonas aeruginosa* iron metabolism and has antimicrobial and antibiofilm activity. *J. Clin. Invest.* 117 877–888. 10.1172/JCI30783 17364024 PMC1810576

[B33] Karmali M. Petric M. Steele B. Lim C. (1983). Sporadic cases of haemolytic-uraemic syndrome associated with faecal cytotoxin and cytotoxin-producing *Escherichia coli* in stools. *Lancet* 321 619–620. 10.1016/S0140-6736(83)91795-6 6131302

[B34] Karmali M. A. (1989). Infection by verocytotoxin-producing *Escherichia coli*. *Clin. Microbiol. Rev.* 2 15–38. 10.1128/CMR.2.1.15 2644022 PMC358098

[B35] Kierek-Pearson K. Karatan E. (2005). Biofilm development in bacteria. *Adv. Appl. Microbiol.* 57 79–111. 10.1016/S0065-2164(05)57003-5 16002010

[B36] Krewulak K. D. Vogel H. J. (2008). Structural biology of bacterial iron uptake. *Biochimica Biophysica Acta* 1778 1781–1804. 10.1016/j.bbamem.2007.07.026 17916327

[B37] Kyle J. L. Parker C. T. Goudeau D. Brandl M. T. (2010). Transcriptome analysis of *Escherichia coli* O157: H7 exposed to lysates of lettuce leaves. *Appl. Environ. Microbiol.* 76 1375–1387. 10.1128/AEM.02461-09 20061451 PMC2832375

[B38] Landstorfer R. Simon S. Schober S. Keim D. Scherer S. Neuhaus K. (2014). Comparison of strand-specific transcriptomes of enterohemorrhagic *Escherichia coli* O157: H7 EDL933 (EHEC) under eleven different environmental conditions including radish sprouts and cattle feces. *BMC Genomics* 15:353. 10.1186/1471-2164-15-353 24885796 PMC4048457

[B39] Lawlor M. S. O’connor C. Miller V. L. (2007). Yersiniabactin is a virulence factor for *Klebsiella pneumoniae* during pulmonary infection. *Infect. Immun.* 75 1463–1472. 10.1128/IAI.00372-06 17220312 PMC1828572

[B40] Lynch M. F. Tauxe R. V. Hedberg C. W. (2009). The growing burden of foodborne outbreaks due to contaminated fresh produce: risks and opportunities. *Epidemiol. Infect.* 137 307–315. 10.1017/S0950268808001969 19200406

[B41] Mantelin S. Touraine B. (2004). Plant growth-promoting bacteria and nitrate availability: impacts on root development and nitrate uptake. *J. Exp. Bot.* 55 27–34. 10.1093/jxb/erh010 14623902

[B42] Marshall K. Stout R. Mitchell R. (1971). Mechanism of the initial events in the sorption of marine bacteria to surfaces. *Microbiology* 68 337–348.

[B43] Matkovich S. J. Zhang Y. Van Booven D. J. Dorn G. W. (2010). Deep mRNA sequencing for in vivo functional analysis of cardiac transcriptional regulators. *Circ. Res.* 106 1459–1467. 10.1161/CIRCRESAHA.110.217513 20360248 PMC2891025

[B44] Meng J. Doyle M. (1997). Emerging issues in microbiological food safety. *Annu. Rev. Nutr.* 17 255–275. 10.1146/annurev.nutr.17.1.255 9240928

[B45] Miethke M. Marahiel M. A. (2007). Siderophore-based iron acquisition and pathogen control. *Microbiol. Mol. Biol. Rev.* 71 413–451. 10.1128/MMBR.00012-07 17804665 PMC2168645

[B46] Murphy K. C. Campellone K. G. (2003). Lambda Red-mediated recombinogenic engineering of enterohemorrhagic and enteropathogenic *E. coli*. *BMC Mol. Biol.* 4:11. 14672541 10.1186/1471-2199-4-11 PMC317293

[B47] O’brien R. Lindow S. (1989). Effect of plant species and environmental conditions on epiphytic population sizes of *Pseudomonas syringae* and other bacteria. *Phytopathology* 79 619–627. 10.1094/Phyto-79-619

[B48] Oh Y. Jo W. Yang Y. Park S. (2007). Influence of culture conditions on *Escherichia coli* O157: H7 biofilm formation by atomic force microscopy. *Ultramicroscopy* 107 869–874. 10.1016/j.ultramic.2007.01.021 17544218

[B49] Olakanmi O. Britigan B. E. Schlesinger L. S. (2000). Gallium disrupts iron metabolism of mycobacteria residing within human macrophages. *Infect. Immun.* 68 5619–5627. 10.1128/IAI.68.10.5619-5627.2000 10992462 PMC101514

[B50] Park Y. Kim Y. (1996). Study on the nutrient composition of hydroponic water dropwort (*Oenanthe stolonifera* DC). *Food Chem.* 57 79–79. 10.1016/0308-8146(96)89021-2

[B51] Perez-Llamas C. Lopez-Bigas N. (2011). Gitools: analysis and visualisation of genomic data using interactive heat-maps. *PLoS One* 6:e19541. 10.1371/journal.pone.0019541 21602921 PMC3094337

[B52] Porcheron G. Garénaux A. Proulx J. Sabri M. Dozois C. M. (2013). Iron, copper, zinc, and manganese transport and regulation in pathogenic Enterobacteria: correlations between strains, site of infection and the relative importance of the different metal transport systems for virulence. *Front. Cell. Infect. Microbiol.* 3:90. 10.3389/fcimb.2013.00090 24367764 PMC3852070

[B53] Raman B. Nandakumar M. Muthuvijayan V. Marten M. R. (2005). Proteome analysis to assess physiological changes in *Escherichia coli* grown under glucose-limited fed-batch conditions. *Biotechnol. Bioeng.* 92 384–392. 10.1002/bit.20570 16180237

[B54] Reizer J. Ramseier T. M. Reizer A. Charbit A. Saier M. H. Jr. (1996). Novel phosphotransferase genes revealed by bacterial genome sequencing: a gene cluster encoding a putative N-acetylgalactosamine metabolic pathway in *Escherichia coli*. *Microbiology* 142 231–250. 10.1099/13500872-142-2-231 8932697

[B55] Risso D. Ngai J. Speed T. P. Dudoit S. (2014). Normalization of RNA-seq data using factor analysis of control genes or samples. *Nat. Biotechnol.* 32 896–902. 10.1038/nbt.2931 25150836 PMC4404308

[B56] Robinson M. D. Oshlack A. (2010). A scaling normalization method for differential expression analysis of RNA-seq data. *Genome Biol.* 11:R25. 10.1186/gb-2010-11-3-r25 20196867 PMC2864565

[B57] Schenk P. M. Carvalhais L. C. Kazan K. (2012). Unraveling plant–microbe interactions: can multi-species transcriptomics help? *Trends Biotechnol.* 30 177–184. 10.1016/j.tibtech.2011.11.002 22209623

[B58] Schikora A. Carreri A. Charpentier E. Hirt H. (2008). The dark side of the salad: *Salmonella typhimurium* overcomes the innate immune response of *Arabidopsis thaliana* and shows an endopathogenic lifestyle. *PLoS One* 3:e2279. 10.1371/journal.pone.0002279 18509467 PMC2386236

[B59] Schneider B. L. Hernandez V. J. Reitzer L. (2013). Putrescine catabolism is a metabolic response to several stresses in *Escherichia coli*. *Mol. Microbiol.* 88 537–550. 10.1111/mmi.12207 23531166 PMC3633691

[B60] Schneider B. L. Kiupakis A. K. Reitzer L. J. (1998). Arginine catabolism and the arginine succinyltransferase pathway in *Escherichia coli*. *J. Bacteriol.* 180 4278–4286. 9696779 10.1128/jb.180.16.4278-4286.1998 PMC107427

[B61] Schneider B. L. Reitzer L. (2012). Pathway and enzyme redundancy in putrescine catabolism in *Escherichia coli*. *J. Bacteriol.* 194 4080–4088. 10.1128/JB.05063-11 22636776 PMC3416515

[B62] Schroeder A. Mueller O. Stocker S. Salowsky R. Leiber M. Gassmann M. (2006). The RIN: an RNA integrity number for assigning integrity values to RNA measurements. *BMC Mol. Biol.* 7:3. 10.1186/1471-2199-7-3 16448564 PMC1413964

[B63] Shi X. Namvar A. Kostrzynska M. Hora R. Warriner K. (2007). Persistence and growth of different *Salmonella serovars* on pre-and postharvest tomatoes. *J. Food Prot.* 70 2725–2731. 10.4315/0362-028X-70.12.2725 18095423

[B64] Sivapalasingam S. Friedman C. R. Cohen L. Tauxe R. V. (2004). Fresh produce: a growing cause of outbreaks of foodborne illness in the United States, 1973 through 1997. *J. Food Prot.* 67 2342–2353. 10.4315/0362-028X-67.10.2342 15508656

[B65] Skaar E. P. (2010). The battle for iron between bacterial pathogens and their vertebrate hosts. *PLoS Pathog.* 6:e1000949. 10.1371/journal.ppat.1000949 20711357 PMC2920840

[B66] Soncini F. C. Véscovi E. G. Groisman E. A. (1995). Transcriptional autoregulation of the *Salmonella typhimurium* phoPQ operon. *J. Bacteriol.* 177 4364–4371. 10.1128/jb.177.15.4364-4371.1995 7543474 PMC177185

[B67] Sperandio V. Nguyen Y. (2012). Enterohemorrhagic *E. coli* (EHEC) pathogenesis. *Front. Cell. Infect. Microbiol.* 2:90. 10.3389/fcimb.2012.00090 22919681 PMC3417627

[B68] Srey S. Jahid I. K. Ha S.-D. (2013). Biofilm formation in food industries: a food safety concern. *Food Control* 31 572–585. 10.1016/j.foodcont.2012.12.001

[B69] Stülke J. Hillen W. (1999). Carbon catabolite repression in bacteria. *Curr. Opin. Microbiol.* 2 195–201. 10.1016/S1369-5274(99)80034-4 10322165

[B70] Taormina P. J. Beuchat L. R. Slutsker L. (1999). Infections associated with eating seed sprouts: an international concern. *Emerg. Infect. Dis.* 5 626–634. 10.3201/eid0505.990503 10511518 PMC2627711

[B71] Tatusov R. L. Koonin E. V. Lipman D. J. (1997). A genomic perspective on protein families. *Science* 278 631–637. 10.1126/science.278.5338.631 9381173

[B72] Tchieu J. H. Norris V. Edwards J. S. Saier M. H. Jr. (2001). The complete phosphotransferase system in *Escherichia coli*. *J. Mol. Microbiol. Biotechnol.* 3 329–346. 11361063

[B73] Tweeddale H. Notley-Mcrobb L. Ferenci T. (1998). Effect of slow growth on metabolism of *Escherichia coli*, as revealed by global metabolite pool (“metabolome”) analysis. *J. Bacteriol.* 180 5109–5116. 9748443 10.1128/jb.180.19.5109-5116.1998 PMC107546

[B74] Vikram A. Jesudhasan P. R. Jayaprakasha G. Pillai B. Patil B. S. (2010). Grapefruit bioactive limonoids modulate *E. coli* O157: H7 TTSS and biofilm. *Int. J. Food Microbiol.* 140 109–116. 10.1016/j.ijfoodmicro.2010.04.012 20471125

[B75] Visca P. Leoni L. Wilson M. J. Lamont I. L. (2002). Iron transport and regulation, cell signalling and genomics: lessons from *Escherichia coli* and *Pseudomonas*. *Mol. Microbiol.* 45 1177–1190. 10.1046/j.1365-2958.2002.03088.x 12207687

[B76] Wu Y. Outten F. W. (2009). IscR controls iron-dependent biofilm formation in *Escherichia coli* by regulating type I fimbria expression. *J. Bacteriol.* 191 1248–1257. 10.1128/JB.01086-08 19074392 PMC2631988

[B77] Yoo W. Kim D. Yoon H. Ryu S. (2017). Enzyme IIANtr Regulates *Salmonella* invasion Via 1, 2-Propanediol and propionate catabolism. *Sci. Rep.* 7:44827. 10.1038/srep44827 28333132 PMC5363084

