# Supplementary material for: Transcriptomic Analysis of Shiga Toxin-Producing Escherichia coli FORC_035 Reveals the Essential Role of Iron Acquisition for Survival in Canola Sprouts and Water Dropwort
Source: Front Microbiol. 2018 Oct 8;9:2397. doi: 10.3389/fmicb.2018.02397 (PMC6186786; doi:10.3389/fmicb.2018.02397)
Supplement: Supplementary file 1 [file Presentation_1.PDF]

Supplementary Material

Transcriptomic analysis of shiga toxin-producing *Escherichia coli* FORC_035 reveals the essential role of iron acquisition for survival in canola sprouts and water dropwort

Hongjun Na[a,†], Yeonkyung Kim[a,†], Dajeong Kim[a,†], Hyunjin Yoon[b], and Sangryeol Ryu[a,c,*]

[a]Department of Food and Animal Biotechnology, Department of Agricultural Biotechnology, Research Institute of Agriculture and Life Sciences, Seoul National University, Seoul 08826, Republic of Korea

[b]Department of Molecular Science and Technology, Department of Applied Chemistry and Biological Engineering, Ajou University, Suwon 16499, Republic of Korea

[c]Center for Food and Bioconvergence, Seoul National University, Seoul 08826, Republic of Korea

*Corresponding author: Sangryeol Ryu (E-mail: sangryu@snu.ac.kr)

[†]These authors contributed equally to this work.

Table S1. Oligonucleotides used in this study.

Primer	Sequence (5'-3')	Source or Reference
**Mutational analysis**		
entB-Lambda-F	AGG CTT ACG CAC TGC CGG AGT CTC ACG ATA TTC CTG TAG GCT GGA GCT GCT TCG	This study
entB-Lambda-R	GAG AGT AGC TTC CAC CAG GCG TCG ATG GTC GGG TTA TTC CGG GGC TCC GTC GAC C	This study
entB-Confirm-F	CGA AAA AAG CTG CGC TTA TC	This study
entB-Confirm-R	GGT TAC CCA GAC ATT TTT ACC	This study
ybtS-Lambda-F	GCA GAA GAA GAG TGT TAT GTC TAT GAG CGT CAA CCC TGT TGG TAT TTA GGC AAA GGG TGC CTG TAG GCT GGA GCT GCT TCG	This study
ybtS-Lambda-R	CGC ATC AAA GCG CGT ATC GTC CAG CAG CAG GAT TGC GCC GGA ATA AAG CTC TCG ATT CCG GGG CTC CGT CGA CC	This study
ybtS-Confirm-F	CAA ATT ACC ACC ACC TCC AC	This study
ybtS-Confirm-R	TCG GCA AGA GAA CTG ATG AC	This study
k1	CAG TCA TAG CCG AAT AGC CT	(Datsenko and Wanner, 2000)
entB-F-pUHE	AAA GGA TCC ATG GCT ATT CCA AAA TTA CAG G	This study
entB-R-pUHE	AAA AAG CTT TTA TTT CAC CTC GCG GGA GA	This study

Table S2. Number of sequence reads in RNA-Seq.

Statistical content	Control (No plant)				Canola sprouts				Water dropwort			
	1 h		3 h		1 h		3 h		1 h		3 h	
	1st	2nd	1st	2nd	1st	2nd	1st	2nd	1st	2nd	1st	2nd
**rRNA reads (%)**	120,176<br>(0.16)	463,954<br>(0.55)	106,774<br>(0.21)	36,988<br>(0.06)	59,860<br>(0.07)	127,572<br>(0.17)	62,820<br>(0.13)	2,333,082<br>(5.41)	62,696<br>(0.08)	47,348<br>(0.06)	63,862<br>(0.18)	23,272<br>(0.04)
**Uniquely mapped reads (%)**	39,100,064<br>(52.05)	45,980,680<br>(54.07)	27,918,382<br>(54.29)	31,211,022<br>(53.79)	48,775,096<br>(59.61)	35,661,656<br>(47.02)	22,604,304<br>(48.52)	18,779,878<br>(43.57)	42,737,088<br>(53.21)	39,757,208<br>(51.82)	18,143,366<br>(51.25)	24,339,398<br>(45.90)
**Multiple mapped reads (%)**	26,722,770<br>(35.58)	28,072,122<br>(33.01)	14,708,226<br>(28.60)	17,309,932<br>(29.83)	22,311,400<br>(27.27)	26,010,020<br>(34,30)	16,012,496<br>(34.37)	14,232,578<br>(33.02)	24,309,470<br>(30.27)	23,984,164<br>(31.26)	10,957,430<br>(30.95)	19,245,558<br>(36.29)
**Unmapped reads (%)**	3,697,876<br>(4.92)	4,456,178<br>(5.24)	3,825,564<br>(7.44)	4,252,980<br>(7.33)	4,255,474<br>(5.20)	6,473,546<br>(8.54)	3,634,540<br>(7.80)	5,687,426<br>(13.20)	6,195,480<br>(7.71)	6,121,454<br>(7.98)	2,823,834<br>(7.98)	4,274,010<br>(8.06)
**QC dropped reads (%)**	5,474,706<br>(7.29)	6,068,222<br>(7.14)	4,865,640<br>(9.46)	5,209,794<br>(8.98)	6,421,444<br>(7.85)	7,565,320<br>(9.98)	4,276,498<br>(9.18)	2,065,960<br>(4.79)	7,011,812<br>(8.73)	6,808,516<br>(8.87)	3,410,784<br>(9.64)	5,147,326<br>(9.71)
**Total reads (%)**	75,115,592<br>(100)	85,041,156<br>(100)	51,424,586<br>(100)	58,020,716<br>(100)	81,823,274<br>(100)	75,838,114<br>(100)	46,590,658<br>(100)	43,098,924<br>(100)	80,316,546<br>(100)	76,718,690<br>(100)	35,399,276<br>(100)	53,029,564<br>(100)

Table S4. Comparison of normalization methods for RNA-Seq.

	Normalization method		
	**RPKM**	**RLE**	**TMM**
**Coefficients of variation (CV)**	0.4681	0.4374	0.4361

Table S5. Primers used in qRT-PCR.

Primer	Locus tag	Sequence (5’-3’)
GAPDH-F	FORC35_3041	TCC GTG CTG CTC AGA AAC G
GAPDH-R	FORC35_3041	CAC TTT CTT CGC ACC AGC G
csgF-F	FORC35_3421	GCT CAG GCC CAA AAC TCT TA
csgF-R	FORC35_3421	CGC GGT TAG CAA TAT CAA CA
fimC-F	FORC35_4551	GCT GGC AGG TAT CCT GAT GT
fimC-R	FORC35_4551	CCT TTA CAC CAT CGG CAT TT
astA-F	FORC35_3007	GTA TCG AAA GGG CAA TCA AAA C
astA-R	FORC35_3007	CAA TGC GTT ATA GAC ATT CAG C
puuC-F	FORC35_2443	AGC AAA GGG CAA CTG TTG TT
puuC-R	FORC35_2443	CGC CTG TTC TTC TGA TGT GA
narU-F	FORC35_2686	TTA CAA CGC CTG CAT CTC TG
narU-R	FORC35_2686	CGC CGA ACT TAT CGG AAA TA
sgcX-F	FORC35_4566	TGG TGC CAA AGA CAA AGA CG
sgcX-R	FORC35_4566	CGA AGC CAC CAG ATA AAC GG
ytfQ-F	FORC35_4683	AGG CGA AAG ATG CCG AAA TC
ytfQ-R	FORC35_4683	AGC CTT TCT TGC GGT CAA TG
ybtS-F	FORC35_2144	GAA CAA TGG CTA CCG ACG AT
ybtS-R	FORC35_2144	AAT AAA TGT CGC CTG CGT TC
fepA-F	FORC35_3890	TTA TAC TCG CTC GAA ATA TGG C
fepA-R	FORC35_3890	CAT CAC GTC ATC AAG ATC GAT A
entB-F	FORC35_3876	GAT TAT TAC CGG GGT ATA TGC C
entB-R	FORC35_3876	TAA TTC TTC AGT CAT CAC CAC C
feoA-F	FORC35_0323	CAC TCC AGA TAC TGC GTG GA
feoA-R	FORC35_0323	ACG GGT TTC GAT ATG AAT GG
efeO-F	FORC35_3440	AGG TGA CCG TGA CCG ATA AG

efeO-R	FORC35_3440	AAA TTC GCC GTC ATT TTC TGG C
exbB-F	FORC35_0739	TAA TGC AGA CGG ACC TTT CC
exbB-R	FORC35_0739	GTT TAA GGA ACG CGC TTC AG
fepD-F	FORC35_3882	ATT GGT CTG CTG GCG ATT AC
fepD-R	FORC35_3882	AGG GGT AGC AAG TAG CGT GA
fhuA-F	FORC35_4284	GGG ACG CTA CGA TTG GTC TA
fhuA-R	FORC35_4284	GAA AGC ACT CGG TTC AAA GG
fecA-F	FORC35_4614	AGG GGT TCT ACA CCC AAA CC
fecA-R	FORC35_4614	GGC GGT GTA GTA ACG CAT TT

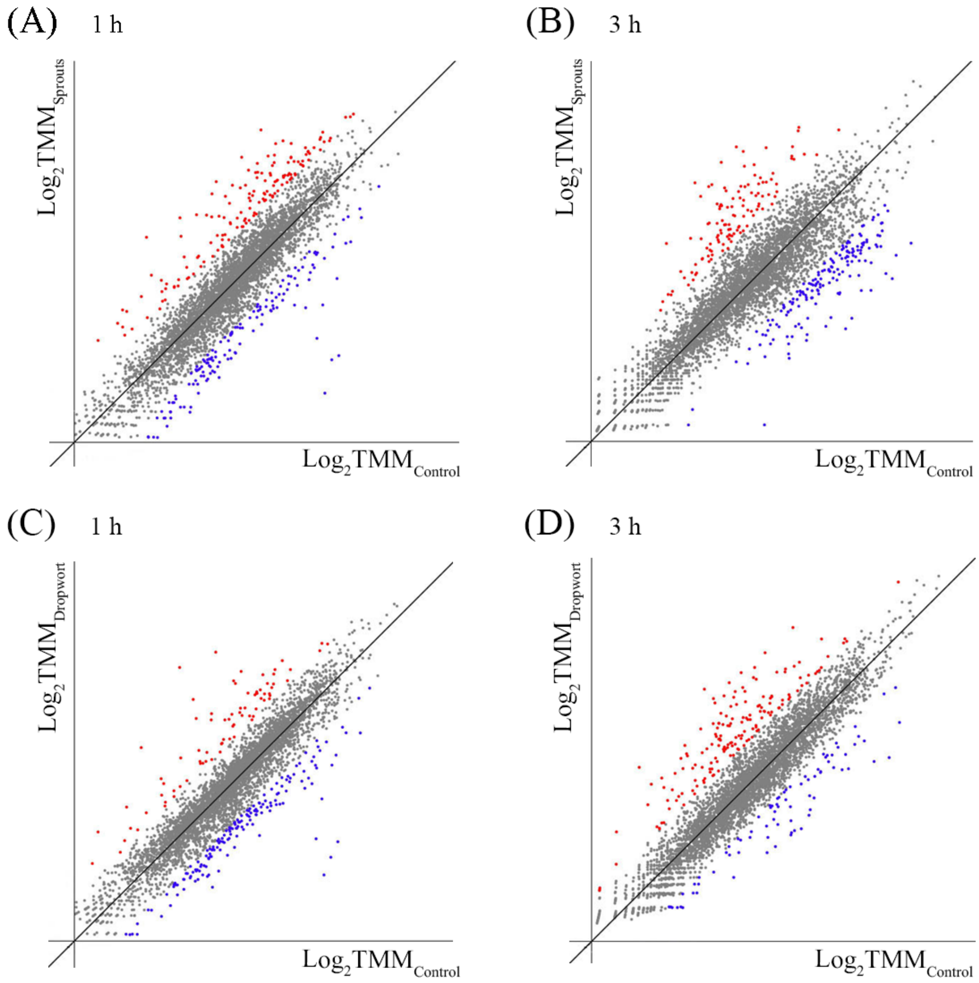

Fig. S1. Analysis of differentially expressed genes (DEGs) in RNA-Seq data. DEGs of FORC_035 in contact with canola sprouts (A and B) and water dropwort (C and D) were identified at 1 h and 3 h post-inoculation by comparing TMM values between plant-exposed and not-exposed samples. The y-axis and x-axis show log-scaled TMM values of genes in contact with fresh produce ($Log_2TMM_{Sprouts}$ or $Log_2TMM_{Dropwort}$) and in no contact ($Log_2TMM_{Control}$), respectively. Red dots indicate up-regulated genes, while blue dots are down-regulated genes, with the criteria of log_2 [fold change] ≥ 2.

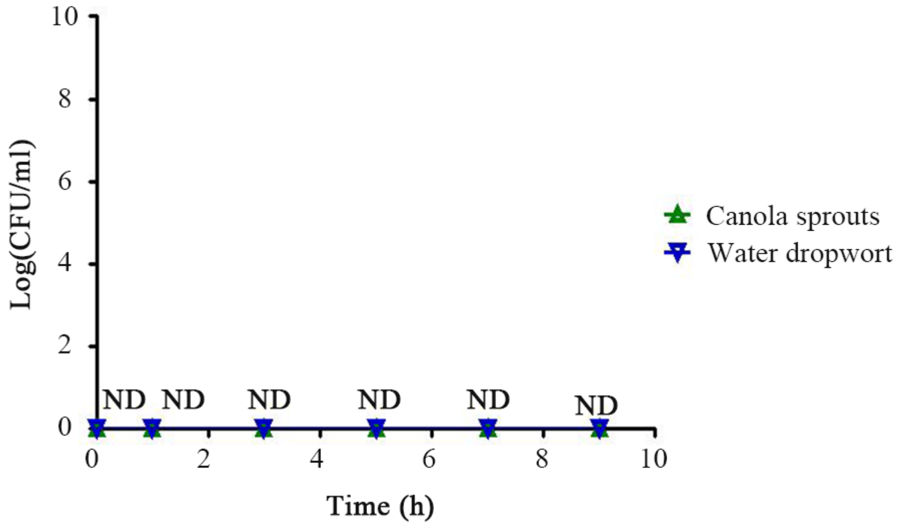

Fig. S2. Growth of *E. coli* indigenous to canola sprouts or water dropwort. Plant tissues were incubated in M9 minimal media without bacterial inoculation. Indigenous *E. coli* living in plants were enumerated by plating the medium broth on TBX agar at each time point. Each symbol indicates the mean value from triplicate measurements. (ND, not detected)

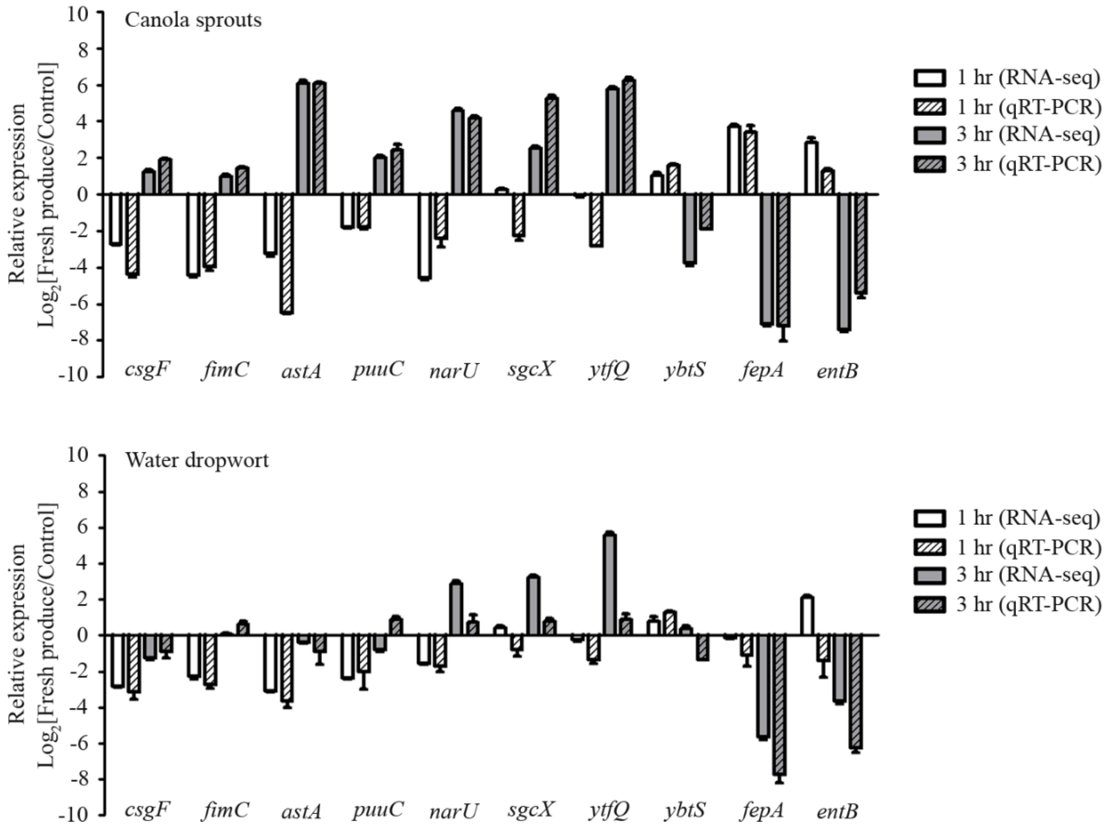

Fig. S3. Validation of differentially expressed genes by qRT-PCR. The RNA-seq results were compared with the relative mRNA levels of the genes measured by qRT-PCR. In qRT-PCR, the mRNA levels were normalized using that of GAPDH. Relative expression was estimated as $\log_2$[Fresh produce/Control] in which control represents expression level of FORC_035 in M9 medium without fresh produce.

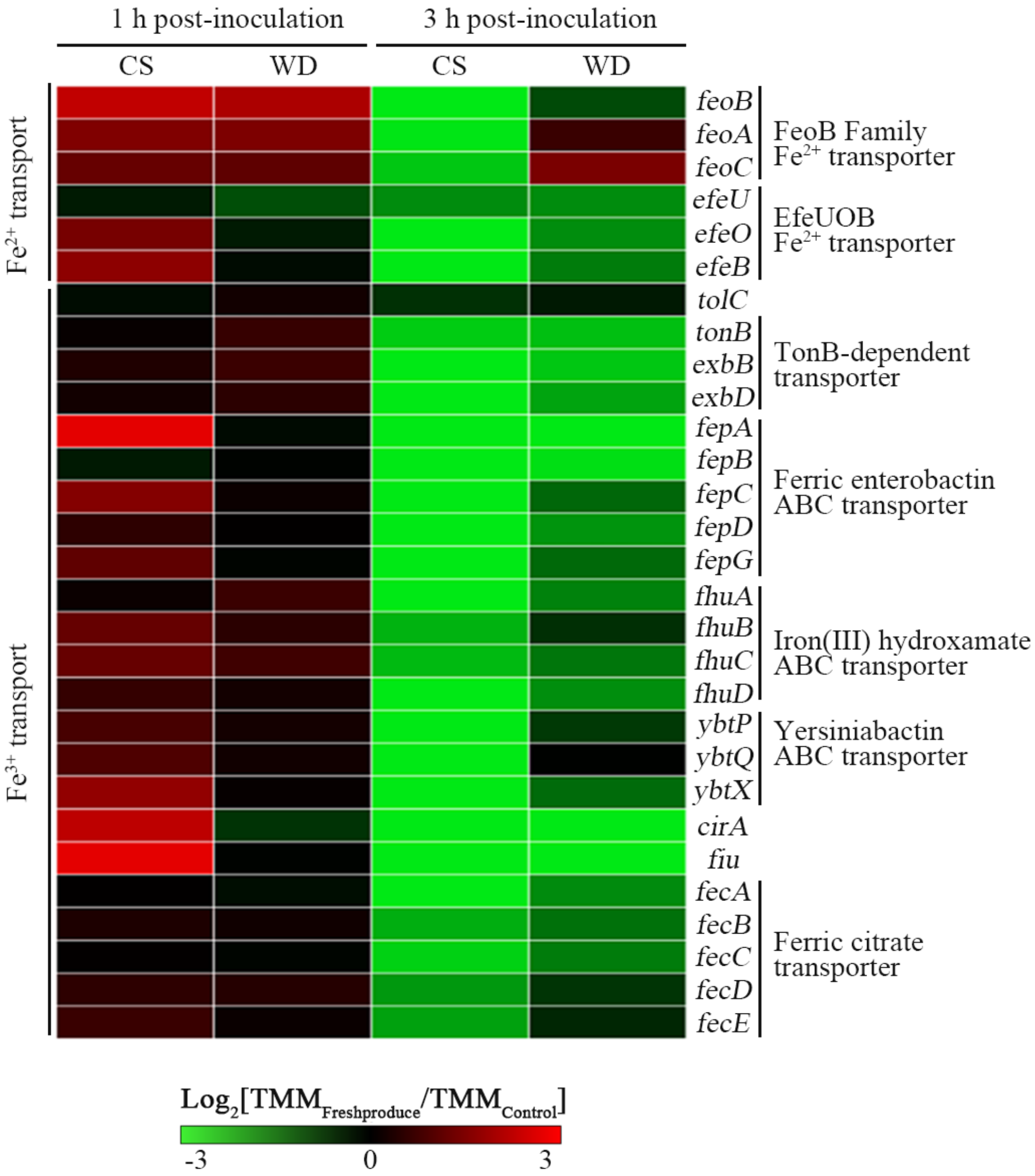

Fig. S4. Heat map of genes involved in iron acquisition. Gene expression ratio was represented using TMM values ($Log_2[TMM_{Fresh\ produce}/TMM_{Control}]$) and depicted using a colorimetric gradient: down-regulation in green and up-regulation in red. (CS, canola sprouts; WD, water dropwort)

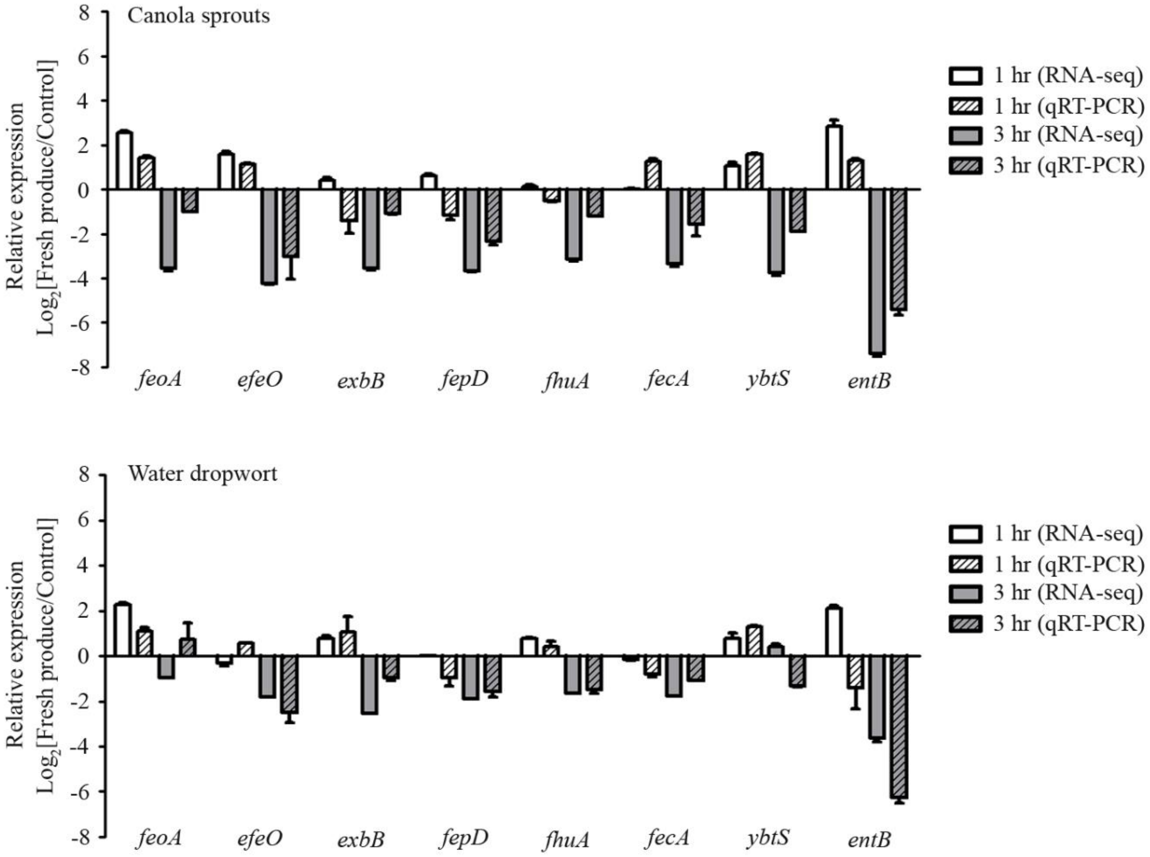

Fig. S5. Validation of genes involved in iron uptake by qRT-PCR. The relative mRNA levels of the genes associated with iron acquisition evaluated by RNA-seq were compared with the results measured by qRT-PCR. In qRT-PCR, the mRNA level was normalized using that of GAPDH. Relative expression was estimated as $\log_2$[Fresh produce/Control] in which control represents expression level of FORC_035 in M9 medium without fresh produce.

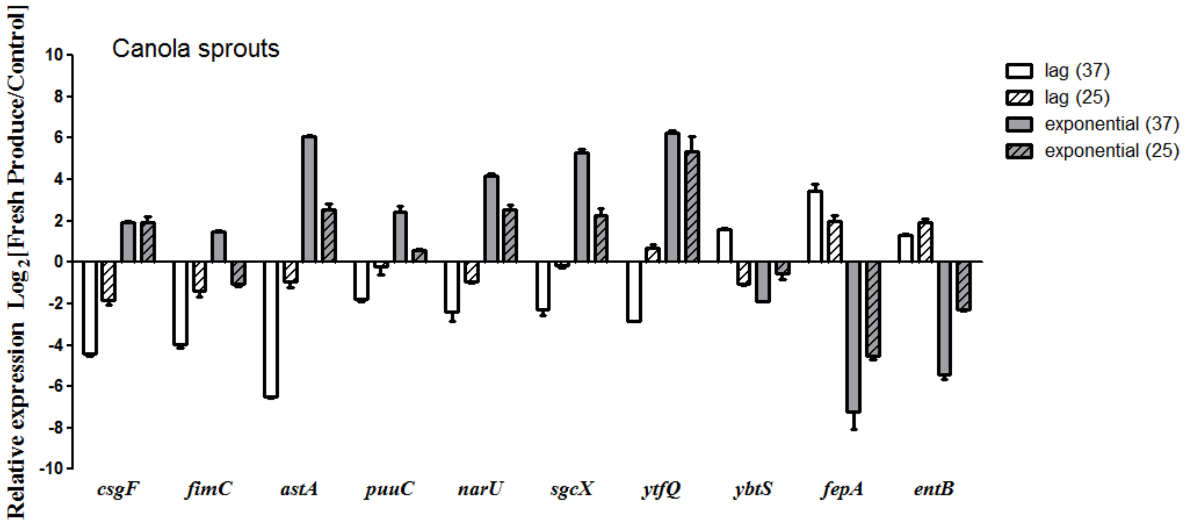

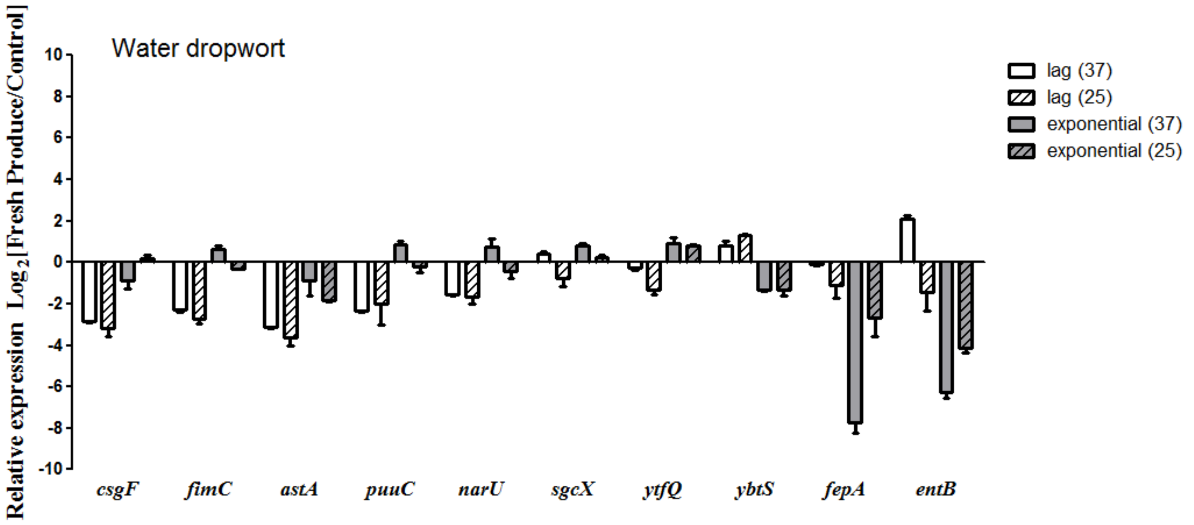

Fig. S6. Comparison of relative expression of representative genes selected from Table 2 of bacterial cells grown at 37°C and 25°C. The relative mRNA levels of the genes were measured by qRT-PCR. The mRNA levels were normalized using that of GAPDH. Relative expression was estimated as $\log_2$[Fresh produce/Control] in which control represents expression level of FORC_035 in M9 medium without fresh produce.

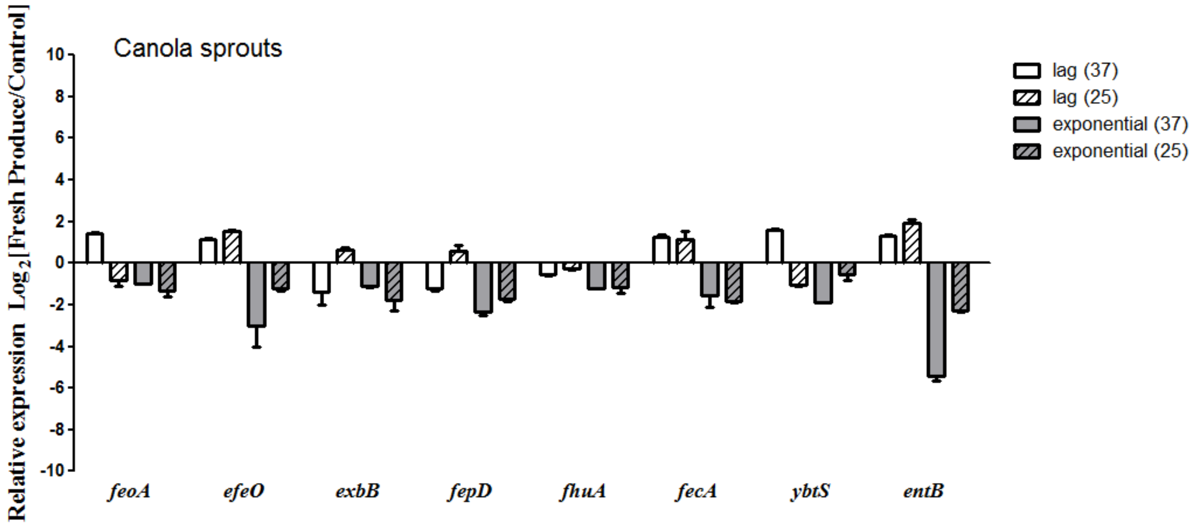

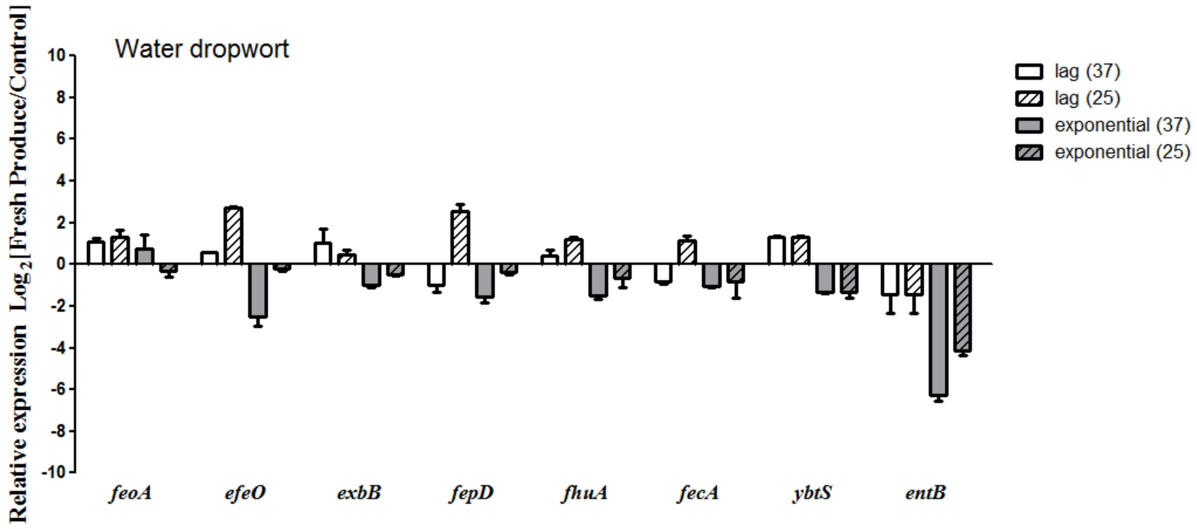

Fig. S7. Comparison of relative expression of genes involved in iron acquisition from bacterial cells grown at 37°C and 25°C. The relative mRNA levels of the genes were measured by qRT-PCR. The mRNA levels were normalized using that of GAPDH. Relative expression was estimated as $\log_2$[Fresh produce/Control] in which control represents expression level of FORC_035 in M9 medium without fresh produce.

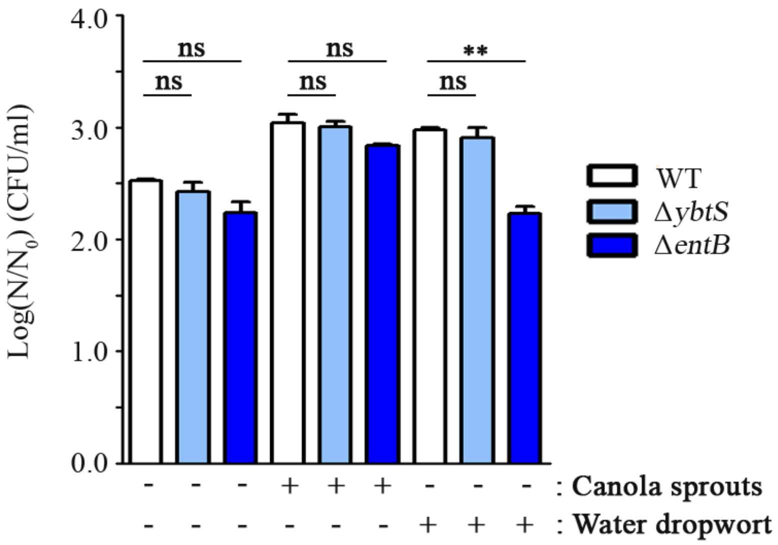

Fig. S8. Comparison of bacterial growth of FORC_035 wild-type (WT), Δ*ybsS*, and Δ*entB* strains. Live bacterial numbers were measured in M9 minimal medium broth supplemented with canola sprouts or water dropwort after a 5-h incubation. Viability of bacterial cells was measured by comparing Log(N/N_0) values, where N_0 is the initial number of cells at 0 h and N is the number of cells after a 5-h incubation. The *t* test was used to evaluate the significance of differences in viability, and significance is indicated as follows: **, $P < 0.01$; ns, not significant.

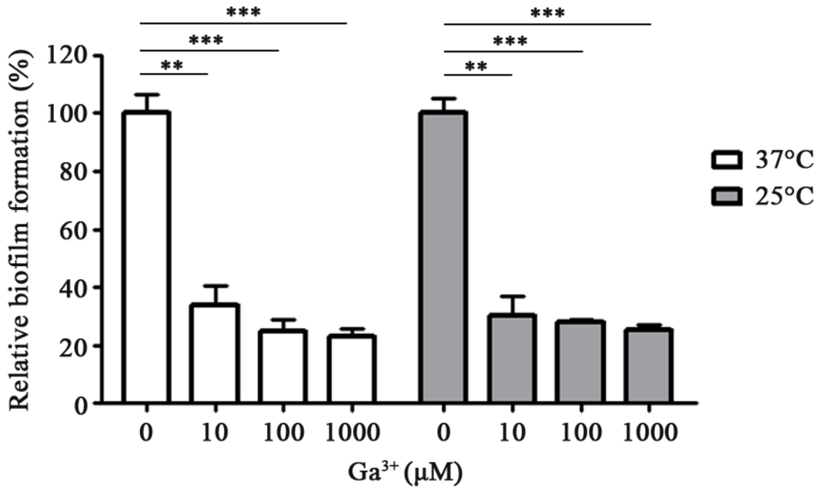

Fig. S9. Gallium inhibits biofilm formation of FORC_035. Biofilm reduction assay was conducted at different temperatures and gallium was added as indicated concentrations. Biofilm was stained using 0.1% crystal violet and OD_{570} was measured. The y-axis represents relative biofilm formation normalized to the value of OD_{570} without gallium treatment, which was set as 100% at each temperature. Asterisks indicate significant differences (**, $P < 0.01$; ***, $P < 0.001$).
